# PSMA-D4 Radioligand for Targeted Therapy of Prostate Cancer: Synthesis, Characteristics and Preliminary Assessment of Biological Properties

**DOI:** 10.3390/ijms22052731

**Published:** 2021-03-08

**Authors:** Piotr Garnuszek, Urszula Karczmarczyk, Michał Maurin, Arkadiusz Sikora, Jolanta Zaborniak, Justyna Pijarowska-Kruszyna, Antoni Jaroń, Monika Wyczółkowska, Wioletta Wojdowska, Dariusz Pawlak, Piotr F. J. Lipiński, Renata Mikołajczak

**Affiliations:** 1 National Centre for Nuclear Research, Radioisotope Centre POLATOM, 05-400 Otwock, Poland; piotr.garnuszek@polatom.pl (P.G.); michal.maurin@polatom.pl (M.M.); arkadiusz.sikora@polatom.pl (A.S.); justyna.pijarowska@polatom.pl (J.P.-K.); antoni.jaron@polatom.pl (A.J.); monika.wyczolkowska@polatom.pl (M.W.); wioletta.wojdowska@polatom.pl (W.W.); dariusz.pawlak@polatom.pl (D.P.); renata.mikolajczak@polatom.pl (R.M.); 2 National Centre for Nuclear Research, 05-400 Otwock, Poland; jolanta.zaborniak@ncbj.gov.pl; 3 Department of Neuropeptides, Mossakowski Medical Research Center Polish Academy of Sciences, 02-106 Warsaw, Poland; plipinski@imdik.pan.pl

**Keywords:** PSMA, DOTA conjugated PSMA ligand (PSMA-D4), prostate cancer, lutetium-177, yttrium-90, scandium-47, actinium-225, in vitro studies, in vivo studies, optical imaging

## Abstract

A new PSMA ligand (PSMA-D4) containing the Glu-CO-Lys pharmacophore connected with a new linker system (L-Trp-4-Amc) and chelator DOTA was developed for radiolabeling with therapeutic radionuclides. Herein we describe the synthesis, radiolabeling, and preliminary biological evaluation of the novel PSMA-D4 ligand. Synthesized PSMA-D4 was characterized using TOF-ESI-MS, NMR, and HPLC methods. The novel compound was subject to molecular modeling with GCP-II to compare its binding mode to analogous reference compounds. The radiolabeling efficiency of PSMA-D4 with ^177^Lu, ^90^Y, ^47^Sc, and ^225^Ac was chromatographically tested. In vitro studies were carried out in PSMA-positive LNCaP tumor cells membranes. The ex vivo tissue distribution profile of the radioligands and Cerenkov luminescence imaging (CLI) was studied in LNCaP tumor-bearing mice. PSMA-D4 was synthesized in 24% yield and purity >97%. The radio complexes were obtained with high yields (>97%) and molar activity ranging from 0.11 to 17.2 GBq mcmol^−1^, depending on the radionuclide. In vitro assays confirmed high specific binding and affinity for all radiocomplexes. Biodistribution and imaging studies revealed high accumulation in LNCaP tumor xenografts and rapid clearance of radiocomplexes from blood and non-target tissues. These render PSMA-D4 a promising ligand for targeted therapy of prostate cancer (PCa) metastases.

## 1. Introduction

Once metastasized, prostate cancer (PCa) becomes one of the most aggressive cancer types. It is the second most common cancer in men and the fifth most common cause of cancer death among men [1,2]. The standard treatment of PCa is based on radical prostatectomy, external beam radiation therapy, or brachytherapy, chemotherapy, and hormonotherapy. Unfortunately, these therapies are often followed by the formation of metastatic castration-resistant prostate cancer (mCRPC) [3].

The PSMA (prostate-specific membrane antigen) known as glutamate carboxypeptidase II (GCP-II) is a transmembrane, 750 amino acid, type II glycoprotein, which is expressed virtually by almost all primary prostate cancer (PCa) and metastatic disease as well [4]. PSMA is highly homologous to N-acetylated R-linked acidic dipeptidase, a neuropeptidase that produces the neurotransmitter glutamate and N-acetylaspartate through the hydrolysis of N-acetylaspartylglutamate [5]. The expression of PSMA is further increased in poorly differentiated, metastatic lesions at the hormone-sensitive and castration-resistant stage and may serve as early progression indicator [6,7,8,9,10,11,12]. Additionally, the increased expression of PSMA in primary PCa correlates with other adverse traditional prognostic factors and independently predicts worse disease outcome [13]. Its expression increases progressively with higher-grade prostate cancer, metastatic disease, and mCRPC [14]. Therefore, PSMA seems to be an ideal target for developing imaging and therapeutic radiopharmaceuticals for PCa.

These findings have spurred the development of PSMA targeting antibodies and small-molecule ligands for radiolabeling with radionuclides for therapeutic use. Initial results have shown that PSMA-targeted radiotherapy can potentially delay disease progression in mCRPC [15]. To date, PSMA has emerged as a potentially accurate and sensitive target for PCa management and has attracted increasing attention as a promising target for PCa imaging and therapy.

Several studies have shown encouraging radiotherapy results with PSMA ligands labeled with beta emitters, including yttrium-90, lutetium-177, iodine-131, and terbium-161. The clinical trials (gov Identifier: NCT04647526) [16], included approximately 800 mCRPC patients treated with PSMA antibodies or PSMA small molecule inhibitors labeled mainly with yttrium-90 and lutetium-177 ([^90^Y]Y-J591, [^177^Lu]Lu-J591, [^177^Lu]Lu-PSMA-617, [^177^Lu]Lu-PSMA I&T, and [^177^Lu]Lu-PSMA-R2) [17,18]. Among the [^177^Lu]Lu-labeled PSMA ligands, the most promising are [^177^Lu]Lu-PSMA-617 and [^177^Lu]Lu-PSMA I&T.

Recent studies have shown that therapy with PSMA ligands labeled with the alpha emitters can overcome resistance to treatment with beta emitters [19,20,21]. To date, the therapeutic efficiency and safety of alpha-targeted therapy of mCRPC have been tested in four clinical studies, including approximately 150 patients treated with PSMA small molecule inhibitor PSMA-617 labeled with actinium-225. Almost 90% of patients have shown prostate-specific antigen (PSA) response in terms of PSA decline with extremely low hematologic toxicity among all treated patients.

The potential utility of PSMA as the target for diagnostic imaging has been clearly shown on the PSMA-11 example [22,23] widely used for ^68^Ga labeling for PET as well as on PSMA-617 [24] and PSMA I&T (Glu-CO-Lys[(Sub)DLys-DPhe-DTyr(3I)-DOTAGA]) [25,26] designed for labeling with a therapeutic radionuclide such as ^177^Lu. At present, [^177^Lu]Lu-PSMA-617 has entered the third phase of clinical trials in patients with progressive PSMA-positive mCRPC.

PSMA-617 (Glu-CO-Lys-2-naphthyl-L-Ala-cyclohexane-DOTA) incorporates Glu-NH-CO-NH-Lys active moiety and two linkers: 2-naphthyl-L-alanine as the first building block and the cyclohexanoic acid connected with DOTA bifunctional chelator (BFC). Such a system is flexible and made the whole molecule show a perfect affinity for PSMA.

It has been presented by Benesova et al. [27] that the pharmacokinetics properties, including PSMA inhibition potencies, cellular internalization, and biodistribution behavior of the PSMA inhibitors, can be significantly influenced by modification of the linker. Thus, the linker moiety’s chemical constitution significantly impacts the in vivo tumor-targeting and pharmacokinetics of PSMA-targeting radioligands.

At National Centre for Nuclear Research, Radioisotope Center POLATOM a novel PSMA ligand, PSMA-T4 (Glu-CO-Lys-L-Trp-4-Amc-HYNIC) (molecular weight (MW) 779.36 g mol^−1^) was developed, which after radiolabeling with ^99m^Tc potentially can be used in the diagnosis of PCa patients (patents pending: EP3721907A1; PL429630A1; US2020324000A1) [28]. The first images in PCa patients obtained with [^99m^Tc]Tc-PSMA-T4 were presented by Sergieva et al. [29,30].

In the development process of the new PSMA ligand, we have focused on using the most appropriate linker system to improve the final radioactive preparation pharmacokinetics. The undertaken studies showed that the presence of L-tryptophan (L-Trp) in PSMA-T4, as one of the linkers, instead of naphthylalanine (L-2NaI), has led to a significant improvement in the biodistribution of the labeled molecule (reducing kidney accumulation) and its increased affinity to PSMA in vivo [28].

Bearing in mind the high affinity of the developed [^99m^Tc]Tc-PSMA-T4 for PSMA in vivo and its confirmed high diagnostic efficacy in patients with PCa metastases, we decided to use this new system, containing two linkers (L-Trp-4-Amc) connected with the Glu-CO-Lys pharmacophore and HYNIC for ^99m^Tc chelation, to develop a new ligand intended for radiolabeling with radionuclides for therapy (i.e., ^177^Lu, ^90^Y, ^47^Sc, and ^225^Ac). For this purpose, we replaced HYNIC with the DOTA as BFC.

This work aims to present synthesis, physicochemical characteristics, and preliminary biological evaluation of a new carrier, PSMA-D4, as a potential candidate for targeted radiotherapy of PCa metastases.

## 2. Results

### 2.1. Synthesis and Physico-Chemical Characteristics of PSMA-D4

**Figure 1:**
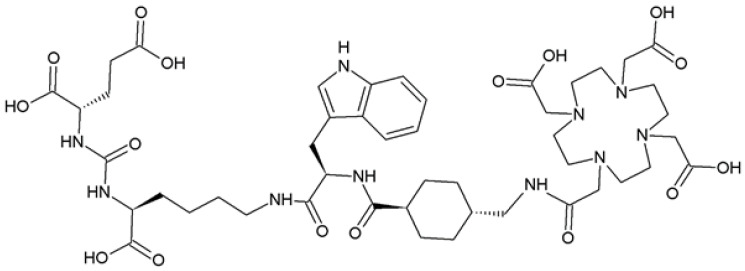
Structure of PSMA-D4 (chemical name: 2-(3-{1-Carboxy-5-[2-({4-[(6-hydrazino-nicotinoylamino)methyl]cyclohexyl}carbonylamino)-3-(1*H*-indol-3-yl)propionylamino]pentyl}ureido)glutaric acid).

PSMA-D4 (Glu-CO-Lys-L-Trp-4-Amc-DOTA, Figure 1) in the form of TFA salt was synthesized following the protocol described herein (Appendix A) and obtained in 24% yield and purity greater than 97%.

**Figure A21:**
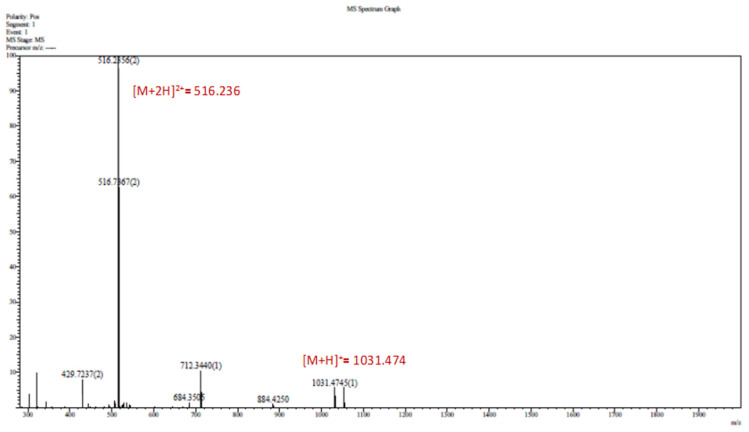
MS spectrum of PSMA-D4.

The identity of PSMA-D4 was confirmed by TOF-ESI-MS and ^1^H, ^13^C-NMR methods (the NMR results are presented in Appendix B). The masses of ions found in MS spectrum (Figure A21, Appendix B) at *m*/*z* [M + 2H]^2+^ = 516.236 and [M + H]^+^ = 1031.474 correspond to the calculated monoisotopic mass of PSMA-D4. The ligand’s net content was 73% calculated based on the C H N elemental analysis (C: 44.04%; H: 5.85%; N: 9.97%).

### 2.2. Molecular Modeling

To understand the structural basis of the observed affinities, compounds PSMA-617, iPSMA-HYNIC (HYNIC-Lys(Nal)-CO-Glu), PSMA-D4, and PSMA-T4 were modeled in complex with glutamate carboxypeptidase II. Initial guesses of the complexes’ structures were built by manual replacement of appropriate elements in the experimental structure of PSMA-1007 with GCPII (PDB accession code: 5O5T [31]). The complexes were then subjected to optimization by local search procedure in AutoDock 4.2.6 [32].

**Figure 2:**
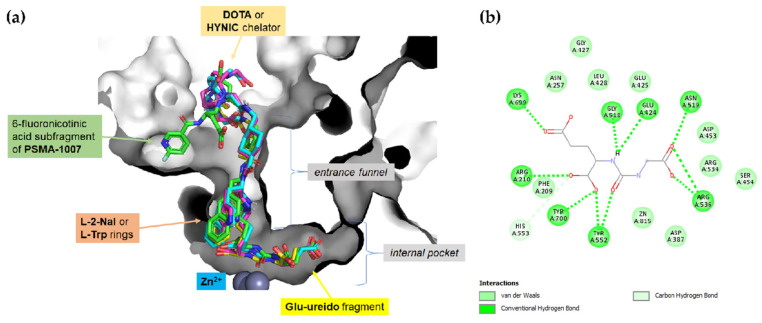
The binding pose of the studied compounds in GCPII: (**a**) general overview in comparison to PSMA1007; (**b**) scheme of the interactions between Glu-ureido fragment and the internal pocket of the enzyme. In part (**a**), the protein is represented as a surface in a simplified manner, and the compounds are shown as colored sticks. Green—PSMA-1007 as found in 5O5T structure [30], light-blue—PSMA-617, yellow—PSMA-T4, and magenta—PSMA-D4.

According to the results of this procedure (general overview in Figure 2a), modifications in either the linker or the chelating element do not influence the contacts of the Glu-ureido pharmacophoric fragment common to all studied compounds and the crystallographic ligand. Thus, Glu-ureido fragment is predicted to be located deep in the GCPII active site (Figure 2b), forming contacts with Arg210, Tyr552, Tyr700, (α-carboxylate of P1’ Glu), Asn257, Lys699 (γ-carboxylate of P1’), Glu424, Gly518 (ureido nitrogens), Tyr552, His553, catalytic Zn^2+^ (ureido carbonyl), Arg534, and Arg536 (P1 carboxylate). These interactions are nearly identical to the ones found for PSMA-1007 in 5O5T structure [31] or those described for other Glu-ureido GCPII binders [33].

**Figure 3:**
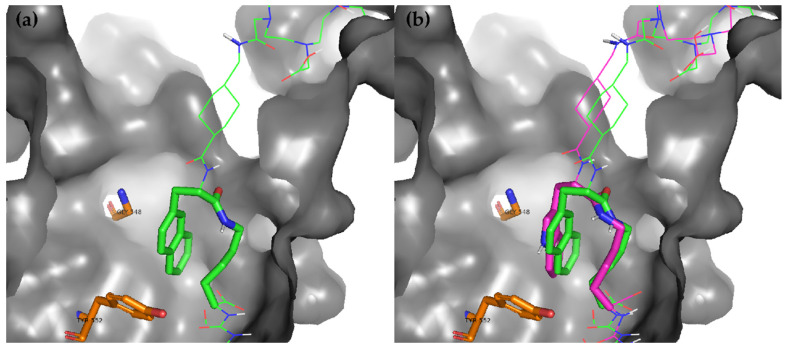
Position of the linker fragment: (**a**) focus on L-2-Nal ring (in (**b**)) superposition of L-2-Nal (in PSMA-617, green) and L-Trp (in PSMA-D4, magenta) rings. The protein is presented as a surface in a simplified manner; additionally, two side chains (orange sticks) are shown.

**Figure A22:**
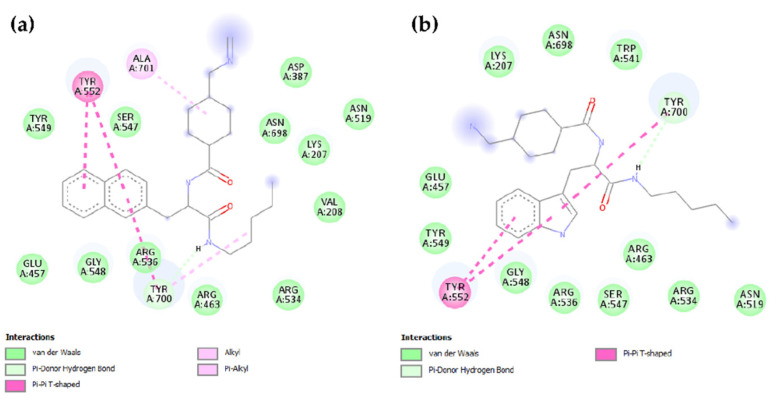
Interactions of the linker part with GCP-II. (**a**) PSMA-617, (**b**) PSMA-D4.

**Figure A23:**
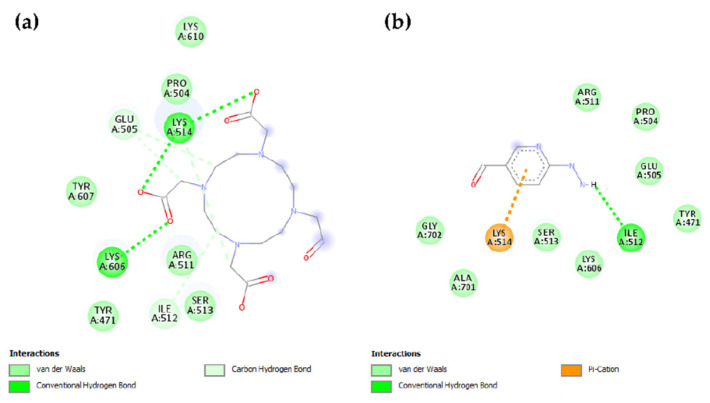
Interactions of the chelator part with GCP-II: (**a**) PSMA-617, (**b**) PSMA-D4.

Regarding the linker part, the naphtyl ring of the alanylnaphtyl residue (in PSMA-617, iPSMA-HYNIC) is predicted to be wedged between the P1 lysine aliphatic chain, Gly548, and Tyr552 (Figure 3a). If the L-2-Nal is exchanged for L-Trp (PSMA-D4, PSMA-T4), docking suggests no significant change in the binding mode. The L-Trp indole ring superposes closely on the position of the napthyl ring in L-2-Nal-bearing analogues (Figure 3b). The 4-aminocyclohexanoic acid fragment leads up the entrance funnel (its position is slightly displaced compared to the crystallographic position of the phenyl ring of PSMA-1007 in 5O5T) to enable the location of DOTA or HYNIC fragments against the residues of helix α15, strand β16, as well as the loop connecting α11 and α12. The chelators are predicted to be involved in several interactions (In Appendix C Figure A22 and Figure A23), but it is to be noted that these interactions might be different in the presence of a chelated cation.

Given the flexibility of both the linker–chelator part of the studied conjugates and the protein by the entrance lid [31], it would be beneficial to inspect the complexes of GCPII with the presented compounds by the use of molecular dynamics.

### 2.3. Radiolabeling of PSMA-D4 and logD

The radiochemical yield (RCY) was always >97% for all radiolabeled compounds and resulted in different specific activity (SA), (Table 1). In case of radiolabeling with ^225^Ac for both used buffers, comparable results of radiochemical purity were observed.

The partition coefficients between n-octanol and PBS (logD) were determined for PSMA-D4 labeled with ^177^Lu and ^90^Y using a shake-flask method. They were compared with the logD values measured under the same experimental conditions for the analogous radiocomplexes of PSMA-617, PSMAI&T labeled with ^177^Lu, and PSMA-11 labeled with ^68^Ga, Table 2. All complexes are highly hydrophilic. The logD values were in the same range for PSMA-D4 and PSMA-617 radiocomplexes, although lower than [^68^Ga]Ga-PSMA-11 (containing the lipophilic HBED-CC chelator) and [^177^Lu]Lu-PSMAI&T.

### 2.4. In Vitro

The competitive binding assay was performed to determine the half-maximal inhibitory concentration (IC_50_) of PSMA inhibitors using cell membranes isolated from lymph node carcinoma of the prostate (LNCaP) cells and [^177^Lu]Lu-PSMA-617 as a radioligand. The IC_50_ values for PSMA-D4 were found to be 28.7 ± 5.2, pointing at their high affinity to PSMA antigen, while PSMA-I&T and PSMA-11 showed significantly lower affinity, and their IC_50_ values were 61.1 ± 7.8 and 84.5 ± 26.5, respectively (Table 3).

The preliminary binding and internalization assays revealed a total binding at the level of 10.2–16.8% with a high internalization (35.4–38.9%) for [^177^Lu]Lu-PSMA-D4, [^90^Y]Y-PSMA-D4, and [^177^Lu]Lu-PSMA-617 with no significant differences between them (Table 4). Only for PSMA-11, the internalization was much lower, at the level of 2.6%.

Saturation radioligand binding assay was performed to determine the affinity (K_D_) of PSMA-D4 labeled with ^177^Lu, ^90^Y, and ^47^Sc for PSMA antigen expressed on LNCaP cell membranes. The cell membranes were incubated with increasing concentration of [^177^Lu]Lu-PSMA-D4, [^90^Y]Y-PSMA-D4, and [^47^Sc]Sc-PSMA-D4 to assess equilibrium dissociation constant (K_D_) value for these compounds. All tested PSMA-D4 radiocomplexes revealed K_D_ value in a low nanomolar range with no significant differences related to the radioisotope used (Table 4). The highest binding affinity observed for [^177^Lu]Lu-PSMA-D4, whose K_D_ value (2.4 ± 0.3 nM) was comparable to [^177^Lu]Lu-PSMA-617 and twice or almost fivefold higher than that for [^177^Lu]Lu-PSMA-I&T and [^68^Ga]Ga-PSMA-11, respectively.

Specificity for PSMA antigen was assessed on LNCaP and human prostate cancer (PC3) cell-lines membranes. Investigated PSMA compounds bound only to the PSMA expressing LNCaP cells, and the specific binding of all tested PSMA-D4 radiocomplexes was within the range of 98.8–99.9% (Appendix D).

### 2.5. Ex Vivo

In that study, ex vivo experiments with [^90^Y]Y-PSMA-D4 and [^47^Sc]Sc-PSMA-D4 were performed in tumor-bearing mice with LNCaP cell-line. Since no binding was observed in in vitro studies with PC-3 cell line, the in vivo studies in mice bearing PC-3 tumors were not performed. Side-by-side comparison of ex vivo distribution is shown in Table 5. Significant differences were observed in the kidneys and tumors. The radiocomplexes were similarly distributed in non-target tissues.

The accumulation of [^90^Y]Y-PSMA-D4 in the tumor was two-fold higher than that of [^47^Sc]Sc-PSMA-D4 at 24 and 48 h after injection, which could be explained by the difference in molar activity of injected complexes (17.2 and 4.57 GBq mcmol^−1^, respectively). The increased tumor uptake led to a high tumor-to-organ ratio. Both radioligands were rapidly cleared from blood circulation by kidneys.

**Figure 4:**
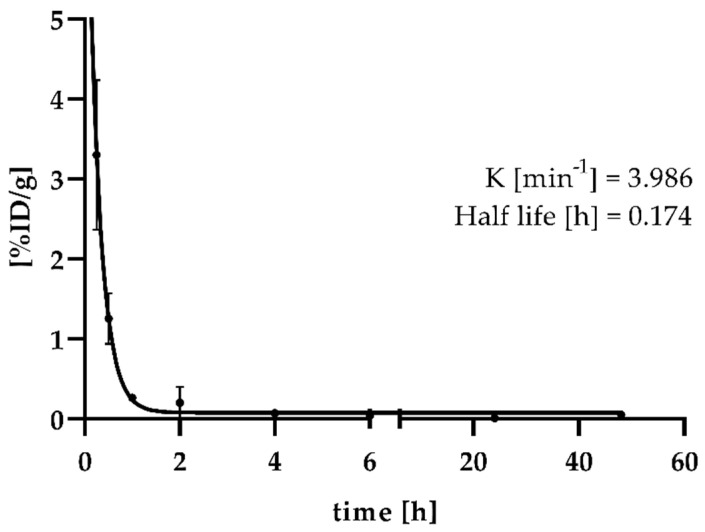
Blood activity clearance of [^47^Sc]Sc-PSMA-D4. The fit of a one-phase model.

The blood pharmacokinetics of [^47^Sc]Sc-PSMA-D4 as a one-phase decay model are shown in Figure 4. Since the complex is rapidly excreted by the kidney, the excretion rate is proportional to the blood plasma concentration.

The radiolabeled [^47^Sc]Sc-PSMA-D4 clears from the blood with a half-life of 0.17 h. We observed a similar one-phase model of blood pharmacokinetics for [^177^Lu]Lu-PSMA-D4 in healthy rats, characterized by the half-life 0.55 [h] (data not presented).

### 2.6. In Vivo

**Figure 5:**
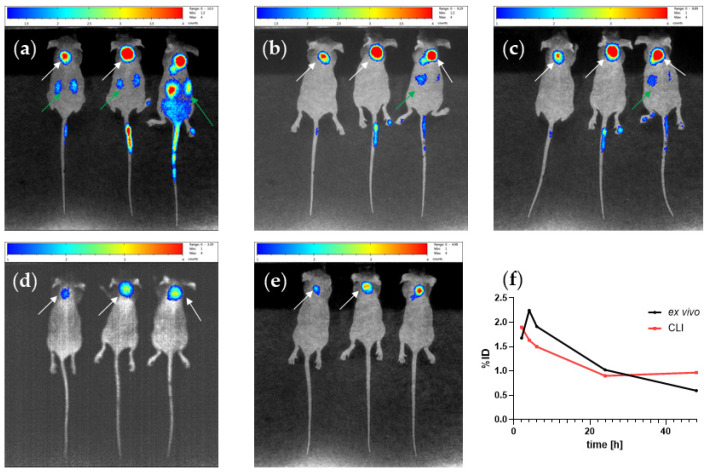
In vivo biodistribution of [^90^Y]Y-PSMA-D4 (**a**–**e**) in LNCaP tumor-bearing mouse model on right shoulder. Representative CLI images at 2, 4, 6, 24, and 48 h p.i., and their comparison with %ID from ex vivo studies (**f**). White arrows indicate the LNCaP, green arrows the kidneys.

Figure 5 shows the CLI of LNCaP tumor-bearing mice at different time points after injection of [^90^Y]Y-PSMA-D4.

Due to the rapid excretion of [^90^Y]Y-PSMA-D4 with urine, radioactivity accumulation in the urinary bladder was not observed. Moreover, in vivo, CLI revealed excellent tumor visualization and clearance of radiolabeled compound from kidneys and the whole body. These observations suggest that the linker system containing L-Trp favorably modifies pharmacokinetics and affinity of the designed ligand for PSMA positive tumors. Rapid clearance of radioactivity from the kidney may reduce toxicity towards this critical organ.

The tumor uptake, calculated as %ID (per cent of injected dose) from in vivo studies, was comparable to that from ex vivo biodistribution studies (Figure 5f). Uptake measurements were done considering regions of interest (ROI’s) corresponding to the tumor and the 0.1 mL of [^90^Y]Y-PSMA-D4 as a standard (injected dose), placed in a black 24-well plate filled with gelatin solution. To refine the calculation of %ID, measurements considering background were done. The background was defined as regions near the heart and well without tracer.

## 3. Discussion

Radionuclide therapy with specific ligands targeting the PSMA inhibitor is a promising therapeutic strategy for patients with mCRPC. Several PSMA ligands and radionuclides have been developed and selected for this purpose. Among them, PSMA-617 and PSMA-I&T labeled with ^177^Lu are the most common in clinical use. The utility significance of ^225^Ac [35] and ^47^Sc is also growing. PSMA therapies have strong potential for a subset of patients and have reached late stage trials; however, the exact patient populations and degree of health benefits in a greater population remain to be determined. On the other hand, despite the high efficacy of the current radioligands used for this purpose, there is always a need to improve treatment outcomes and minimize side effects, including using newer agents with more favorable therapeutic properties.

Bearing in mind the importance of each element of a potential isotope carrier molecule, including the linker connecting the bioactive part of the drug to the radionuclide [27] in the molecular targeting of the molecule to the binding site, we have developed a new ligand for targeted radiotherapy of mCRPC. From our previous studies on the PSMA ligand for ^99m^Tc labeling [28], we found that the presence of the L-Trp fragment in the linker system significantly reduces the accumulation of the radioactive product in the kidney, which in the case of a therapeutic formulation is of great importance due to the reduction of radiotoxicity towards this organ. Replacement of HYNIC in the developed PSMA-T4 molecule with DOTA (PSMA-D4) did not result in the loss of this property but reduced the renal affinity while maintaining a high affinity for the PSMA inhibitor.

In silico molecular modeling and docking studies of the new PSMA-D4 molecule to GCPII showed the high similarity of its binding mode to that of PSMA-617. When comparing to published data on PSMA-617 [36], the in vitro properties of PSMA-D4 are largely the same. These observations included n-octanol/PBS distribution ratio and K_D_ and internalization in PSMA-positive and PSMA-negative cancer cells. In vitro studies also confirmed promising properties of a PSMA-D4 when labeled with therapeutic radionuclides.

Ex vivo studies demonstrated that [^90^Y]Y-PSMA-D4 and [^47^Sc]Sc-PSMA-D4 accumulated favorably in LNCaP tumor-bearing mice immediately after injection and rapid elimination with the urine. Key findings from these observations include low accumulation in the kidney, relatively fast clearance of radioactivity from blood and non-target tissues, and a high tumor-to-background ratio. These circumstances enable Cerenkov luminescence imaging, which confirmed the tumor-to-background contrast is increasing over time.

Radioactivity uptake in selected organs of investigated PSMA-ligands of LNCaP and PC-3 PIP (PSMA positive) tumor bearing mice is summarized in Table 6.

The summary of radioligand accumulation in PSMA-positive tumors and critical organs in Table 6 allows the conclusion that labeled PSMA-D4 shows favorable affinity properties in vivo. However, it is difficult to make a quantitative assessment in comparison to other radioligands due to the variability as to the used experimental models, as well as due to the administered masses of the preparations.

## 4. Materials and Methods

### 4.1. Chemicals and Radionuclides

Polystyrene-based Wang resin, all amino acids, and coupling reagents—N,N’-diisopropylcarboxydiimide, OxymaPure, COMU, and PyOxim were purchased from Iris Biotech GmbH (Marktredwitz, Germany). DOTA(tBu)_3_ was purchased from CheMatech (Dijon, France). The purity of amino acids and chelators was greater than 98%. PSMA-I&T was purchased from piCHEM (Grambach, Austria). PSMA-617 and PSMA-11 used for experiments were synthesized at RC POLATOM, following the descriptions by Benešová et al. [36] and Eder et al. [23].

All other reagents were purchased either from Iris Biotech GmbH (Nordost, Germany) or Sigma-Aldrich (Steinheim am Albuch, Germany). Solvents were purchased from Avantor Performance Materials Poland S.A. (Gliwice, Poland) or Sigma-Aldrich (Germany). Solvents used for HPLC and LC-MS were purchased from VWR Chemicals (Radnor, PA, USA) or Sigma-Aldrich. L(+)-Ascorbic acid was purchased from PanReac AppliChem, (cat. no. 141013.1211, Darmstadt, Niemcy).

Lutetium-177 (LutaPol) as lutetium chloride of SA higher than 555 MBq mg^−1^ Lu in 0.04 N HCl and yttrium-90 (ItraPol) as a yttrium chloride in a 0.04–0.05 N HCl of 0.925–37 GBq in a volume 0.010–2 mL were produced at Radioisotope Centre POLATOM, (Otwock, Poland). Scandium-47 was produced via the nuclear reaction ^46^Ca(n,γ)^47^Ca→^47^Sc, by irradiation of enriched ^46^Ca target at the Institute Laue-Langevin (Grenoble, France) or Maria research reactor (Otwock, Poland), and ^47^Sc was separated from the irradiated target by extraction chromatography on DGA resin [41]. Actinium-225 as actinium nitrate was purchased from the Institute of Physics and Power Engineering (Obninsk, Russia). Gallium-68 as a gallium chloride solution in 0.1 M HCl was obtained from the GalliaPharm^®^ (Ge-68/Ga-68 generator manufactured by Eckert & Ziegler (Dresden, Germany).

### 4.2. Synthesis of PSMA-D4

**Scheme 1:**
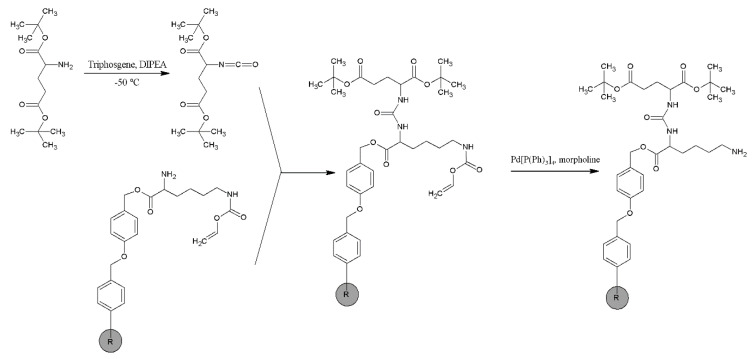
Solid-phase synthesis of the Glu(tBu)-urea-Lys-NH2 moiety.

The PSMA-D4 synthesis was performed by standard solid-phase synthesis from the Fmoc-L-Lys(Alloc) attachment to the Wang polystyrene resin. Preparation of Glu(tBu)-urea-Lys-NH_2_ was carried according to Scheme 1.

**Scheme 2:**
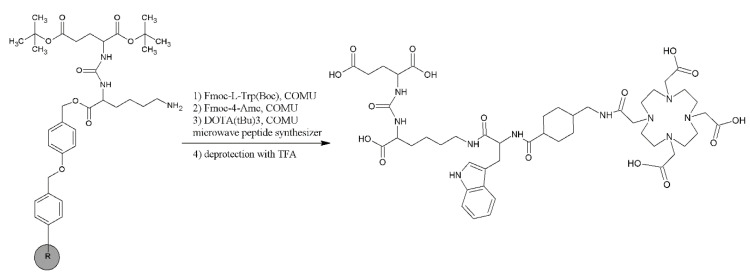
Coupling of the L-tryptophane, 4-Amc, and DOTA chelator to the Glu(tBu)-urea-Lys-NH2 moiety and final deprotection.

Further steps of synthesis carried on solid support were performed in an automatic peptide synthesizer LibertyBlue equipped with Discover microwave oven (CEM) [42,43] according to Scheme 2.

The 1-[(1-(cyano-2-ethoxy-2-oxoethylideneaminooxy)dimethylaminemorpholino)]uronium hexafluorophosphate (COMU) was used as a coupling agent, with N,N-diisopropylethylamine as a base [44].

The product was detached from the resin and deprotected, based on the publication of Wängler et al. [45].

The raw PSMA-D4 (Glu-CO-Lys-LTrp-4Amc-DOTA) was purified on preparative HPLC on a reversed-phase column, resulting in >98% purity peptide.

### 4.3. Analytical Methods

#### 4.3.1. HPLC

Analytical HPLC was performed using a Shimadzu system consisting of LC-20AD pump, SPD-M20A diode array detector (DAD), CBM-20A controller, and the LC Solution software (Shimadzu Europa GmbH, Duisburg, Germany).

HPLC conditions were as follows: column: Phenomenex Kinetex C18, 5 µm, 150 × 4.6 mm column, flow rate: 1 mL min^−1^, solvent: 80% of 0.1% TFA in water and 20% of 0.1% TFA in acetonitrile, oven temp. 40 °C. The UV/Vis acquisition wavelength was set in the range of 190–300 nm.

#### 4.3.2. LC-MS

LC-MS analysis was run on Shimadzu HPLC Prominence system equipped with mass spectrometer LCMS-IT-TOF (Shimadzu Europa GmbH, Duisburg, Germany) system consisting of electrospray source (ESI), ion trap (IT), time of flight analyzer (TOF), and the LCMS Solution software. Samples were analyzed using the same HPLC analytical method as described above. Conditions of mass spectrometry analysis were as follows: ionization: electrospray; ionization mode: positive; interference voltage: 5.0 kV; CDL temperature: 270 °C; heat block temperature: 270 °C; nebulizing gas flow: 1.5 L min^−1^; mass range m/z: 200–1500 Da; and ion accumulation: 100 ms.

The identity of PSMA-D4 was confirmed by the mass spectrometry method with the use of ESI-IT-TOF detector. The peptide sample was dissolved in a mixture of AcN/water and, after injection, was ionized by the electro spray method. The molecular mass of the sample was determined in a positive mode.

#### 4.3.3. ^1^H, ^13^C-NMR

The NMR spectra were recorded at Varian VNMRS-600 spectrometers equipped with a 5-mm PFG AutoXID(^1^H/X^15^N-^31^P) probe. The sample was dissolved in 0.6 mL of DMSO-D6—99.80% D Euriso-top lot: Q22681, batch: 0817H.

The structure of the PSMA-D4 was determined by interpretation of the one-dimensional ^1^H, ^13^C, and DEPT-135 spectra, two-dimensional homonuclear COSY, TOCSY, and ROESY and heteronuclear ^1^H-13C HSQC, HSQC-TOCSY, and HMBC NMR spectra. Proton connectivities were derived from COSY, TOCSY, and ROESY spectra. The ^13^C resonances corresponding to carbons with directly attached protons were assigned using HSQC and HSQC-TOCSY spectra. HMBC spectra were used to assign resonances of the quaternary carbons and to validate the connectivities established by the other spectra. Results of both ^1^H-^15^N correlations (HSQC and HMBC) were used to confirm nitrogen atoms’ character in the compound studied.

The details of the NMR analysis are presented in Appendix B.

#### 4.3.4. Elemental Analysis

The percentage content of C, H, and N was analyzed in an automatic UNIcube analyzer. The measurement’s basis was catalytic combustion of the analyzed substance in a special pipe, with oxygenation, at 1150 °C.

Combustion gas was separated from each other on adsorption columns and determined successively using a thermal conductivity detector (TCD).

#### 4.3.5. Lipophilicity Determination

The partition coefficient logD of the radioligands was determined by the shake-flask method [46,47]. The solution of 10 to 50 MBq of radiolabeled PSMA ligands (D-4, 617, 11, I&T) in 1.0 mL of phosphate-buffered saline (PBS, pH 7.4) was added to 1.0 mL of presaturated n-octanol solution (*n* = 3). Vials were shaken vigorously for 10 min. To achieve quantitative phase separation, the vials were centrifuged at 1600 rpm for 5 min. The radioactivity concentration in a defined volume of both the aqueous and the organic phase in six replicates each was measured in a γ-counter (Wizard 1470, Wallac, Turku, Finland). The partition coefficient was calculated as the logarithm of the ratio between counts per minute (cpm) measured in the n-octanol phase to the PBS phase counts.

### 4.4. Docking Methodology

Modeling of glutamate carboxypeptidase II (GCP-II) complexes with the compounds PSMA-617, iPSMA-HYNIC, PSMA-D4, and PSMA-T4 was performed in a two-stage manner.

The starting point was the crystallographic structure of GCP-II with PSMA-1007 (PDB accession code: 5O5T [31]). In the first stage, the vector–linker substructure of the considered compounds was built by removing and replacing appropriate elements from PSMA-1007 (in the linker part). The linker was capped with the acetyl group. The complexes were subject to local search docking with AutoDock 4.2.6 [32] with the following parameters: 150 individuals in a population, 300 iterations of the Solis–Wets local search, local search space set to 30.0, and 100 local search runs. The results were clustered, and the representative pose of the best scored cluster was taken for further modeling. In this stage, structures of the chelators were built into the complexes.

In the case of DOTA structure, the coordinates of the chelator were taken from the NOJYIU entry [48] of the Cambridge Structural Database (DOTA complex with diaqua-lutetium(III)-sodium trihydrate) in order to maintain the conformation found with a chelated metal present. Then, vector–linker–chelator position was optimized again using local search docking with AutoDock 4.2.6 [32] with the following parameters: 150 individuals in a population, 300 iterations of the Solis–Wets local search, local search space set to 45.0, and 500 local search runs. The procedure was repeated several times to check convergence of the results. Additionally, the DOTA fragment was modeled with the flexibility of the carboxylate arms enabled or not, and with protonated and unprotonated state of this group, but upon finding no major differences in the poses, only the results of dockings with unprotonated, flexible carboxylates are discussed.

The receptor structure was prepared in AutoDockTools [32]. The box was set around the experimental position of the crystallographic ligand and extended. The grids were calculated with AutoGrid 4. Molecular graphics were prepared in the Discovery Studio Visualizer [49] and PyMOL [50].

### 4.5. Radiolabeling and QC

Radiolabeling with ^177^Lu and ^90^Y was carried out in ascorbic acid sodium salt solution (pH 4.5–5.0) containing PSMA ligand (depending on the experiment 100 mcg PSMA-D4 or 70 mcg PSMSA-617 or 50 mcg PSMA I&T) and 5–20 mcL of [^177^Lu]LuCl_3_ or [^90^Y]YCl_3_ (100–700 MBq), up to 0.5 mL. Reaction mixtures were incubated at 95 ± 5 °C for 15 min.

Radiolabeling with ^47^Sc was carried out in ascorbic acid sodium salt solution (pH 4.5–5) containing 50 mcg of PSMA-D4 and 800 mcL of [^47^Sc]ScCl_3_ (100–250 MBq), up to 1.5 mL. Reaction mixtures were incubated at 95 ± 5 °C for 20 min.

Radiolabeling with ^225^Ac was carried out in 0.1 M TRIS buffer (pH 9) or ascorbic acid sodium salt solution (pH 4.5–5) containing 100 mcg of PSMA-D4 and 50 mcL of [^225^Ac]AcNO_3_ (1.85 MBq), up to 0.5 mL. Reaction mixtures were incubated at 95 ± 5 °C for 20 min.

Radiolabeling with ^68^Ga was carried out by adding 5 mL of ^68^Ga eluate (600–800 MBq) to a vial containing 40 mcg of PSMA-11 and 60 mcg sodium acetate. The mixture was incubated at 95 °C for 15 min.

The RCY of the final formulation was determined by thin-layer chromatography on silica-gel plates (ITLC SG) with 0.1 M citric buffer pH 5 as a mobile phase to differentiate between the free and PSMA-D4 bound radionuclide. The radiolabeling yield was evaluated in a competitor’s presence (10 mM DTPA) in excess, which reacts with the non-incorporated radionuclide. In the case of [^225^Ac]Ac-PSMA-D4, after development, the chromatography strips were stored for at least 45 min for the decay of unbound ^221^Fr and to reach radiochemical equilibrium between ^225^Ac and its daughter nuclide ^221^Fr. Subsequently, radiochemical purity was determined by measuring the activity of the γ emission of ^221^Fr. Due to a high RCY of radio conjugates, no additional purification step was necessary.

### 4.6. In Vitro

The LNCaP cell line used for in vitro and in vivo experiments was purchased from American Type Culture Collection (ATTC) and was maintained as per ATCC guidelines. As a control in in vitro studies, the androgen-independent human prostate cancer cells, PC3 (derived from bone metastasis), obtained from National Institute of Medicines (NIL), were used. PC3 cells were cultured in RPMI 1640 medium (IITD PAN Wroclaw, Poland) supplemented with 10% fetal bovine serum (FBS, Gibco) and 1% of penicillin/streptomycin (Gibco). All cells were grown to 80–90% confluence before trypsinization.

To determine cell uptake and internalization of [^177^Lu]Lu-PSMA-D4 and [^90^Y]Y-PSMA-D4, LNCaP cells (5 × 10^5^ cells well^−1^) were incubated in un-supplemented RPMI 1640 medium with one concentration of tested radioligands on 12-well plates in 37 °C per 2 h. After the appropriate time, the medium was removed, and cells were washed twice using phosphate buffer saline (PBS, IITD PAN, Wroclaw, Poland). The cells were treated twice with 1 mL of 50 mM glycine in 0.1 M NaCl at a pH of 2.8 and incubated for 5 min at room temperature to collect extracellularly bound compound. The internalization was determined by measuring the radioactivity of 1mL of 1 M NaOH used for cell lysis. Total binding was calculated as a sum of the membrane and internalized fractions measured using Wallac Wizard 1470 γ counter. As a control, [^177^Lu]Lu-PSMA-617 and [^68^Ga]Ga-PSMA-11 were used.

The equilibrium constant (K_D_) was determined by a saturation binding experiment performed in PSMA positive LNCaP and PSMA negative PC3 cell membranes isolated according to the procedure developed by Fichna et al. [51]. The 50 mcL of LNCaP and PC3 cell membranes were seeded on special MultiScreen™ 96 well filter plates (Merck, Warsaw, Poland) and incubated for 2 h with increasing concentration of [^177^Lu]Lu-PSMA-D4, [^90^Y]Y-PSMA-D4, and [^67^Sc]Sc-PSMA-D4 (0.016–194 nM) at 37 °C. After incubation, the unbound radiocomplexes were filtered under vacuum, and the membranes were washed twice using PBS. The filters containing membranes with the attached radiotracer were extruded into tubes using MultiScreen Multile Punch (Merck). The number of associated inhibitors was determined by measuring the radioactivity of squeezed filters using Wallac Wizard 1470 γ counter. The K_D_ and B_max_ were calculated by nonlinear regression using the GraphPad Prism statistic program.

The half-maximal inhibitory concentration (IC_50_) of PSMA-D4 was determined in a competitive binding assay carried out on 96-well MuliScreen filter plates using LNCaP cell membranes (50 mcL) incubated with [^177^Lu]Lu-PSMA-617 as a radioligand and ten different concentrations (2.4–9708 nM) of PSMA-D4. After 2 h incubation, plates were treated in the same way as in the saturation binding assay. The amount of bound radioactivity was measured in γ-counter. IC_50_ value was evaluated using GraphPad Prism software (Darmstadt, Germany).

### 4.7. Animal Study

This study was conducted following the guidelines approved on 4 July 2018, by the first Local Animal Ethics Committee in Warsaw, Poland (the authorization number 681/2018), and was carried out in accordance with the national legislation regarding laboratory animals’ protection and the principles of good laboratory practice.

Fifty males, 5–6 weeks old BALB/c NUDE mice, were obtained from Janvier Lab., France. Mice were housed under pathogen-free conditions with food and water ad libitum, and a 12–12 h light–dark cycle. Veterinarian staff and investigators observed the mice daily to ensure animal welfare and determine if humane endpoints (e.g., hunched, ruffled appearance, apathy, ulceration, severe weight loss, and tumor burden) were reached.

An experimental tumor murine model was induced using LNCaP cell, which grew to 80–90% confluence before trypsinization and formulation in Matrixgel™ Basement Membrane Matrix (Bedford, MA, USA) for implantation into mice. The mice were subcutaneously injected in the shoulder with 200 mcL bolus containing a suspension of 10^6^ freshly harvested cell line LNCaP in Matrixgel™. This procedure was performed under anaesthesia with 2% isoflurane. The animals were kept under pathogen-free condition, and experiments were performed 2–3 weeks later when a tumor reached a volume of approximately 150 ± 60 mm^3^. Then, the animals were randomized into two groups (25 mice per group, five mice per time point): Group 1, treated with a single intravenous injection of [^90^Y]Y-PSMA-D4 (0.1 mL, 0.1 mcg, 15.5 MBq, SA 16.7 GBq mg^−1^ = 17.2 GBq mcmol^−1^) and Group 2, treated with a single intravenous injection of [^47^Sc]Sc-PSMA-D4 (0.1 mL, 0.19 mcg, 10 MBq, SA 4.8 GBq mg^−1^ = 4.57 GBq mcmol^−1^).

At 1, 2, 4, 6, 24, and 48 h after injection, the animals were euthanized by cervical dislocation and dissected. Selected organs and tissues were assayed for their radioactivity and weighted. The physiological distribution was calculated and expressed in terms of the percentage of administrated radioactivity found in each of the selected organs or tissues per gram (%ID g^−1^).

The pharmacokinetic analysis of the [^47^Sc]Sc-PSMA-D4 was based on the curves of the %ID g^−1^ accumulated in the blood. The pharmacokinetic parameters were determined according to a one-phase decay model. The following parameters were used: K (rate constant) and half-life calculated as ln(2)/K.

### 4.8. Optical Imaging of [^90^Y]Y-PSMA-D4

The PhotonIMAGER Optima system (Biospace Lab, Nesles-la-Vallée, France) was used for the non-invasive detection, localization, and quantification of yttrium-90 signal from live animals based on Cerenkov luminescence imaging. It was feasible due to high-energy *β*^−^ particle emission produces continuous spectrum light photons or Cerenkov radiation. Optical imaging of [^90^Y]Y-PSMA-D4 in LNCaP cell grafted mice was performed contemporaneously with ex vivo studies. The animals were placed individually in an induction chamber, where anaesthesia was induced with 5% isoflurane (Iso-Vet, Piramal Healthcare UK Limited, West Drayton, UK) in 100% oxygen with a delivery rate of 2 L min^−1^ until loss of righting reflex. After induction, the animals were moved to the optical chamber. Anaesthesia was then maintained with 1.5–2% isoflurane in 100% oxygen with a flow of 1.5 L min^−1^ administered through a facemask connected to a coaxial circuit. Body temperature was maintained at 37 °C by a heating table inside the chamber. At recovery, all animals received 100% oxygen until recovery of righting reflex. No mice were restrained during anaesthesia.

### 4.9. Statistics

Results are provided as mean ± SD. The results of physiological distribution are presented as a percentage of the dose administered per gram of tissue (%ID g^−1^) as average value with standard deviation (%ID g^−1^; mean ± standard deviation (SD)) with *n* representing the number of animals per group. Data were statistically analyzed using GraphPad Prism version 8.0.0 for Window.

For blood activity data, a one-phase exponential decay model was used to model the percentage of remaining activity (%ID g^−1^) as a time post-injection function.

Two-tailed, unpaired Student’s *t*-tests assessed results of normal distribution. Otherwise, outcomes were evaluated by a Mann–Whitney U-test. A one-way ANOVA test analyzed the differences between IC_50_ and internalization result. A *p*-value of < 0.05 with two-tailed testing was considered statistically significant.

## 5. Conclusions

In this work, we present a novel PSMA-D4 ligand (Glu-CO-Lys-L-Trp-4-Amc-DOTA), which showed excellent radiolabeling characteristics, high selectivity towards PSMA receptors in vitro, and favorable tumor accumulation in LNCaP tumor-bearing mice. These features render PSMA-D4 a promising ligand for targeted radionuclide therapy of prostate cancer, enriching the state-of-the-art and paving the way to its further development for clinical use. Therefore, extended preclinical studies are planned to determine the pharmacokinetics and toxicity and to confirm the specificity of radio-labeled PSMA-D4 in vivo.

## Figures and Tables

**Table 1:** The radiochemical yield of radiolabeled PSMA-D4 with different radionuclides.

Compound	RCY [%]	SA [GBq mcmol^−1^]	pH
[^177^Lu]Lu-PSMA-D4	99.6 ± 0.1	17.2	4.5–5.0
[^90^Y]Y-PSMA-D4	99.7 ± 0.1	17.2	4.5–5.0
[^47^Sc]Sc-PSMA-D4	99.6 ± 0.1	4.57	4.0–4.5
[^225^Ac]Ac-PSMA-D4	99.3 ± 0.2	0.116	8.5–9.0

**Table 2:** Comparison of lipophilicity of PSMA radiocomplexes.

Compound	logD
[^177^Lu]Lu-PSMA-D4	−5.16 ± 0.07
[^90^Y]Y-PSMA-D4	−5.18 ± 0.04
[^177^Lu]Lu-PSMA-617	−5.12 ± 0.07
[^90^Y]Y-PSMA-617	−5.08 ± 0.13
[^68^Ga]Ga-PSMA-11	−3.46 ± 0.06
[^177^Lu]Lu-PSMA-I&T	−4.47 ± 0.14

**Table 3:** Comparison of IC_50_ of PSMA complexes.

Compound	IC_50_ [nM]	*p*
PSMA-D4	28.7 ± 5.2	-
PSMAI&T	61.1 ± 7.8	0.0059
PSMA-11	84.5 ± 26.5	<0.0001
PSMA-617	2.7 ± 0.1 [34] *	0.0259

[*] Data obtained from Tönnesmann R et al. [34].

**Table 4:** Total binding, internalization ratio, binding affinity (K_D_), and specific binding of PSMA radioligands on human prostate cancer cells and their membranes.

Compound	Total Binding (%)	Internalization (%)	K_D_ (nM)	Specific Binding (%)
[^177^Lu]Lu-PSMA-D4	10.2	37.0 ± 3.3	2.4 ± 0.3	99.1 ± 0.3
[^90^Y]Y-PSMA-D4	16.8	35.4	4.5 ± 2.1	99.9 ± 0.1
[^47^Sc]Sc-PSMA-D4	n.d.	n.d.	3.4 ± 2.0	98.8 ± 0.9
[^177^Lu]Lu-PSMA I&T	n.d.	n.d.	5.1 ± 2.6	97.1 ± 2.9
[^68^Ga]Ga-PSMA-11	13.1	2.6	11.4 ± 7.1	96.8 ± 2.7
[^177^Lu]Lu-PSMA 617	12.8 ± 1.0	38.9 ± 1.7	2.0 ± 0.3 [34] *	n.d.

[*] Data obtained from Tönnesmann R et al. [34].; n.d.—no data.

**Table 5:** Ex vivo biodistribution of [^90^Y]Y-PSMA-D4 and [^47^Sc]Sc-PSMA-D4 in mice bearing LNCaP tumor.

		2 h	4 h	6 h	24 h	48 h
**[^90^Y]Y-PSMA-D4**	blood	0.06 ± 0.01	0.05 ± 0.03	0.06 ± 0.01	0.03 ± 0.03	0.04 ± 0.02
	liver	0.08 ± 0.00	0.08 ± 0.01	0.31 ± 0.07	0.04 ± 0.01	0.17 ± 0.07
	spleen	0.19 ± 0.06	0.17 ± 0.06	1.05 ± 0.29	0.12 ± 0.06	0.97 ± 0.62
	kidneys	2.70 ± 0.20	1.70 ± 0.13	0.98 ± 0.38	0.30 ± 0.12	0.12 ± 0.03
	small intestine	0.32 ± 0.13	0.30 ± 0.14	0.14 ± 0.11	0.20 ± 0.20	0.14 ± 0.07
	large intestine	0.12 ± 0.05	0.32 ± 0.16	0.20 ± 0.02	0.41 ± 0.15	0.84 ± 0.56
	stomach	0.10 ± 0.04	0.11 ± 0.05	0.04 ± 0.03	0.06 ± 0.05	0.03 ± 0.03
	tumor	20.24 ± 5.52	16.40 ± 6.88	8.28 ± 1.97	8.33 ± 3.09	8.03 ± 7.31
	muscle	0.21 ± 0.13	0.05 ± 0.02	0.01 ± 0.01	0.02 ± 0.02	0.02 ± 0.01
	urine [%ID]	96.08 ± 0.53	95.47 ± 3.07	96.57 ± 2.44	94.79 ± 7.51	97.75 ± 0.34
	T/B	323.7	312.9	131.2	257.6	193.0
	T/M	97.0	324.4	983.7	387.4	351.4
	T/K	7.6	9.7	8.4	29.0	68.4
		**1 h**	**4 h**	**6 h**	**24 h**	**48 h**
**[^47^Sc]Sc-PSMA-D4**	blood	0.29 ± 0.08	0.08 ± 0.04	0.04 ± 0.01	0.01 ± 0.01	0.05 ± 0.03
	liver	0.13 ± 0.02	0.08 ± 0.04	0.06 ± 0.01	0.03 ± 0.01	0.04 ± 0.01
	spleen	0.52 ± 0.19	0.57 ± 0.31	0.08 ± 0.04	0.04 ± 0.01	0.10 ± 0.09
	kidneys	9.46 ± 1.44	2.16 ± 0.86	1.42 ± 0.28	0.29 ± 0.09	0.26 ± 0.14
	small intestine	0.35 ± 0.19	0.41 ± 0.30	0.11 ± 0.07	0.34 ± 0.18	0.45 ± 0.13
	large intestine	0.09 ± 0.03	0.70 ± 0.48	0.69 ± 0.17	0.12 ± 0.12	1.63 ± 0.31
	stomach	0.23 ± 0.09	0.39 ± 0.17	0.08 ± 0.09	0.05 ± 0.06	0.03 ± 0.04
	tumor	9.24 ± 5.13	9.22 ± 2.21	8.65 ± 3.19	5.28 ± 3.01	4.11 ± 1.08
	muscle	0.10 ± 0.06	0.39 ± 0.17	0.03 ± 0.02	0.02 ± 0.02	0.06 ± 0.06
	urine [%ID]	89.23 ± 2.63	93.42 ± 2.07	94.89 ± 1.34	97.89 ± 1.22	95.37 ± 1.89
	T/B	31.6	120.9	207.6	467.3	78.8
	T/M	135.2	24.3	251.0	229.0	66.7
	T/K	1.0	4.3	6.1	18.4	16.0

Values are expressed as %ID g^−1^ and presented as mean ± standard deviation. Ratio: T/B—tumor to blood; T/M—tumor to muscle; T/K—tumor to kidneys.

**Table 6:** Biodistribution (%ID g^−1^) of PSMA-ligands in LNCaP tumor-bearing mice (a) and PC-3 PIP (PSMA positive) tumor-bearing mice (b).

a	%ID g^−1^			Time p.i.v.	Dose	Mice	Refs.
	Spleen	Kidneys	Tumor				
[^177^Lu]Lu-PSMA I&T	5.85 ± 2.26	107.24 ± 15.61	7.96 ± 1.76	1 h	0.2 nmol	CD-1 nu/nu	[14]
[^177^Lu]Lu-PSMA-I&T	7.9 ± 2.6	105.3 ± 15.8	8.0 ± 1.7	1 h	0.2 nmol	CD-1 nu/nu	[37]
[^90^Y]Y-PSMA-D4	0.19 ± 0.06	2.70 ± 0.20	20.24 ± 5.52	2 h	0.1 nmol	BALC/c Nude	-
[^47^Sc]Sc-PSMA-D4	0.52 ± 0.19	9.46 ± 1.44	9.24 ± 5,13	1 h	0.2 nmol	BALC/c Nude	-
[^111^In]In-PSMA-I&T	47.4 ± 13.2	207.9 ± 23.9	8.1 ± 1.1	1 h	0.2 nmol	CB17 SCID	[38]
[^68^Ga]Ga-PSMA I&T	3.88 ± 1.46	53.26 ± 9.02	4.95 ± 1.57	1 h	0.2 nmol	CD-1 nu/nu	[14]
[^68^Ga]Ga-PSMA-I&F	12.8 ± 6.5	105.8 ± 22.7	4.5 ± 1.8	1 h	n.d.	SHO	[37]
[^68^Ga]Ga-PSMA-I&T	5.3 ± 1.3	52.6 ± 13.2	5.0 ± 1.6	1 h	0.2 nmol	CB17 SCID	[38]
[^68^Ga]Ga-PSMA-I&T	3.9 ± 1.5	53.3 ± 9.0	4.9 ± 1.6	1 h	n.d.	SHO	[37]
[^68^Ga]Ga-PSMA-11	n.d.	n.d.	7.28 ± 0.82	1 h	n.d.	Nude	[39]
**b**	**%ID g^−1^**			**Time p.i.v.**	**Ddose**	**Mice**	**Refs.**
	**Spleen**	**Kidneys**	**Tumor**				
[^47^Sc]Sc-PSMA-617	0.81 ± 0.34	5.97 ± 0.90	46.7 ± 4.36	2 h	1 nmol	BALB/c Nude	[40]
[^177^Lu]Lu-PSMA-617	0.21 ± 0.03	3.97 ± 0.56	45.8 ± 4.02	2 h	1 nmol	BALB/c Nude	[40]
[^68^Ga]Ga-PSMA-617	0.91 ± 0.14	3.48 ± 0.18	55.8 ± 14.2	2 h	1 nmol	BALB/c Nude	[40]
[^68^Ga]Ga-PSMA-11	2.18 ± 0.38	58.8 ± 7.62	40.0 ± 2.55	2 h	1 nmol	BALB/c Nude	[40]

n.d.—no data

**Scheme A1:**
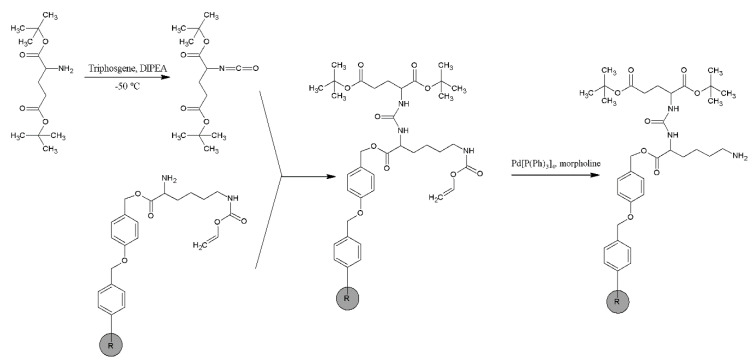
Solid phase synthesis of the Glu(tBu)-urea-Lys-NH_2_ moiety.

**Scheme A2:**
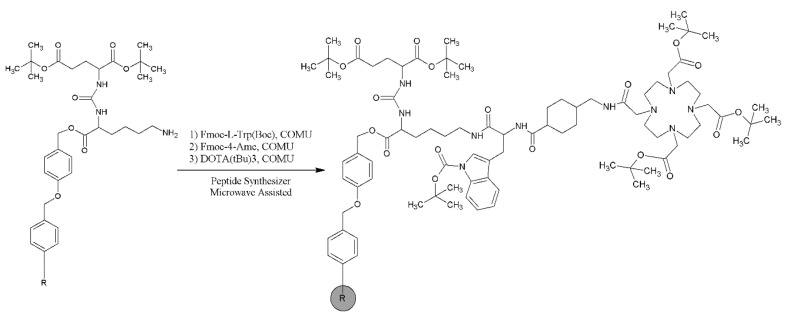
Coupling of the tryptophane, Amc, and DOTA chelator to the Glu(tBu)-urea-Lys-NH2 moiety.

**Scheme A3:**
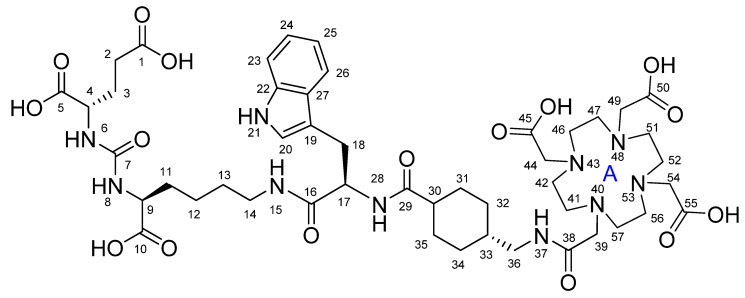
The numbering of PSMA-D4-s15-20 for the 1H/13C/15N.

**Figure A1:**
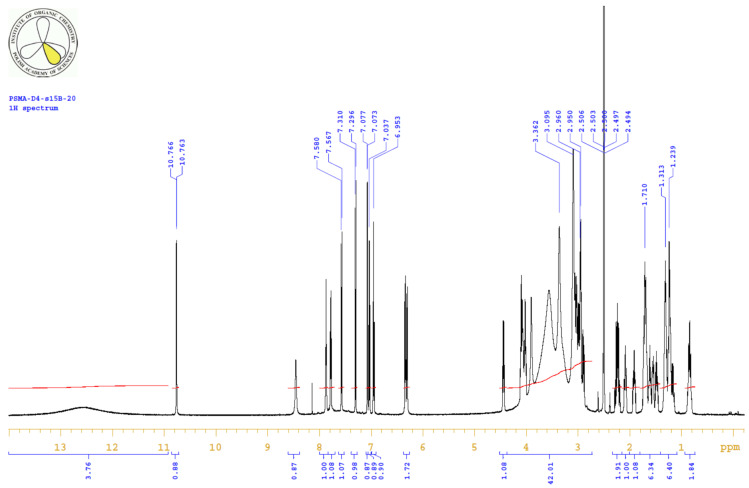
Full ^1^H NMR spectrum of PSMA-D4 in DMSO at 298 K.

**Figure A2:**
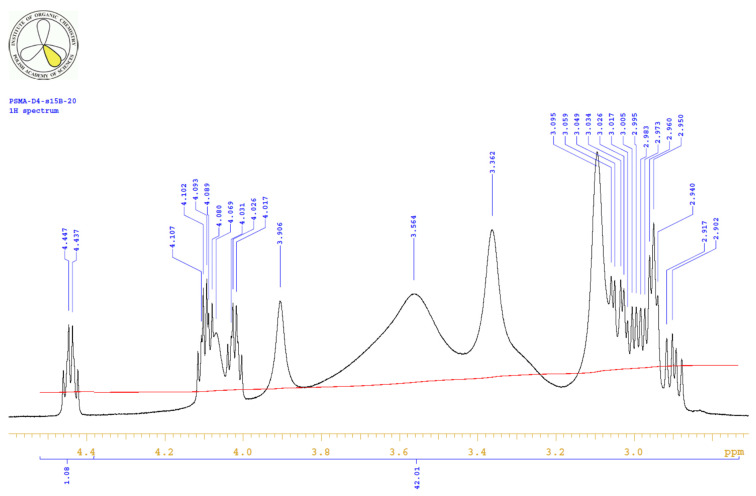
Up-field part of the ^1^H NMR spectrum of PSMA-D4 in DMSO at 298 K.

**Figure A3:**
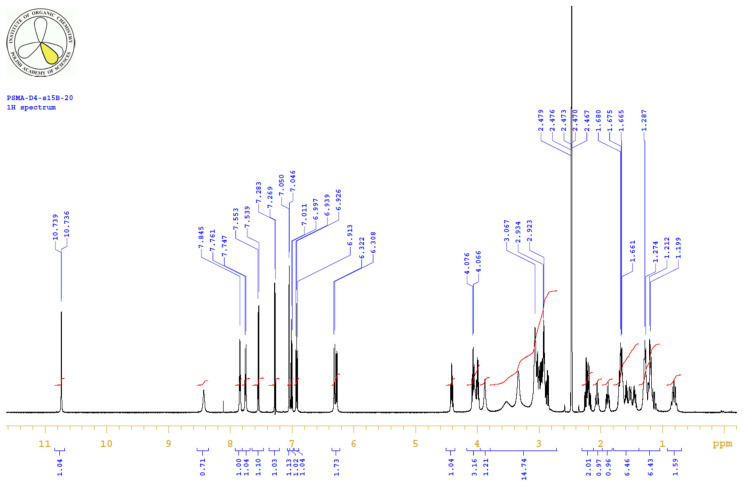
Full ^1^H NMR spectrum of PSMA-D4 in DMSO at 298 K with broad signals elimination sequence.

**Figure A4:**
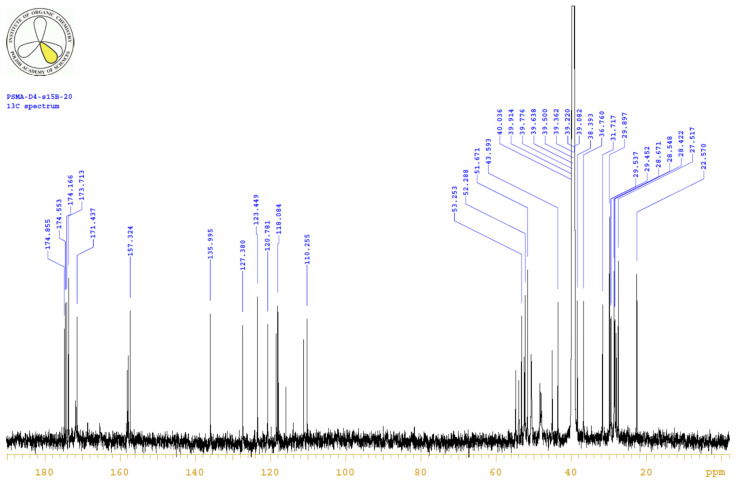
Full ^13^C NMR spectrum of PSMA-D4 in DMSO at 298 K.

**Figure A5:**
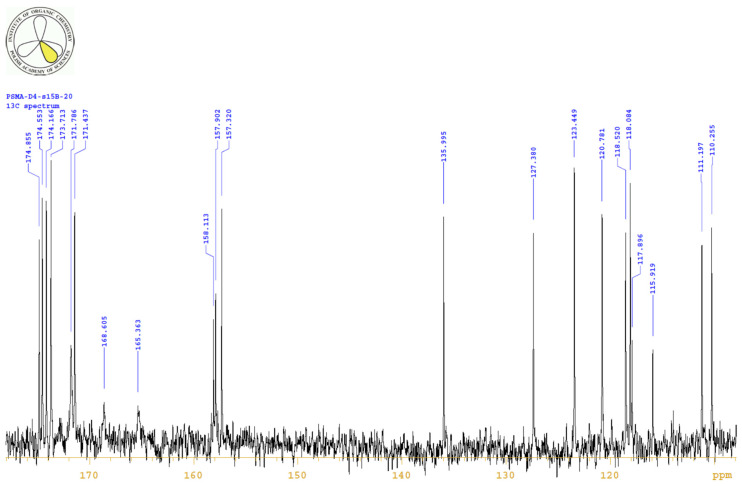
Up-field part of the ^1^H ^13^C NMR spectrum of PSMA-D4 in DMSO at 298 K.

**Figure A6:**
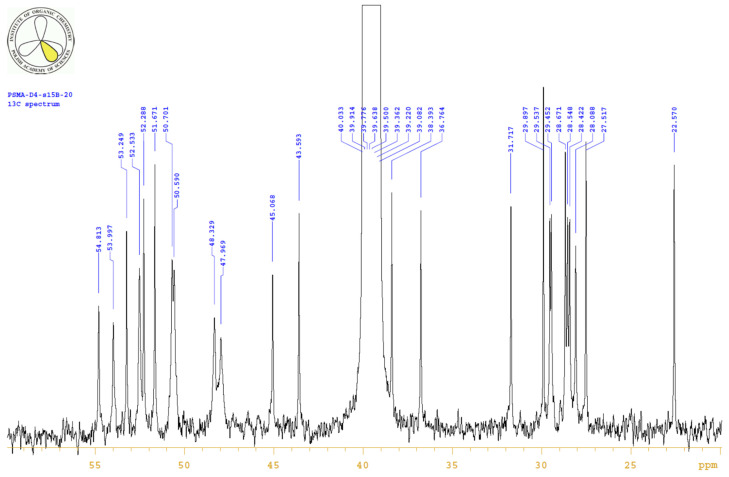
Down-field part of the ^13^C NMR spectrum of PSMA-D4 in DMSO at 298 K.

**Figure A7:**
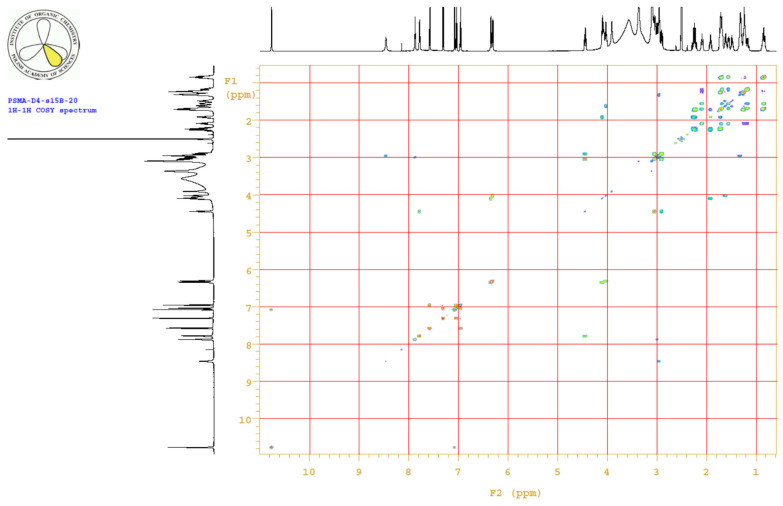
The ^1^H-^1^H COSY spectrum of PSMA-D4 in DMSO at 298 K.

**Figure A8:**
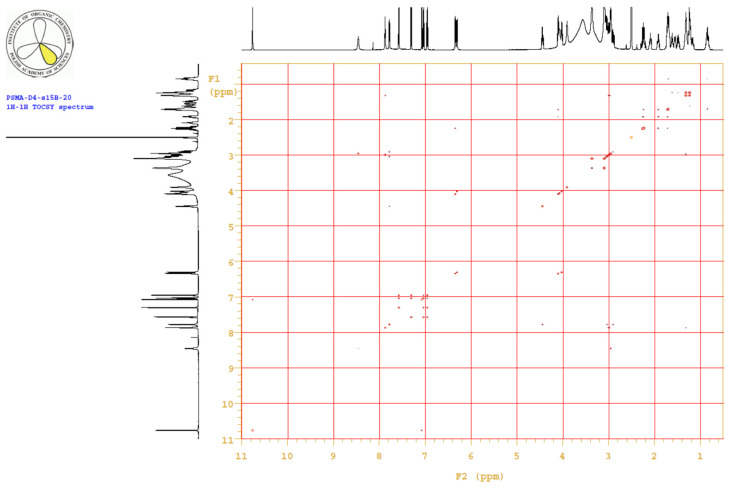
The ^1^H-^1^H TOCSY spectrum of PSMA-D4 in DMSO at 298 K.

**Figure A9:**
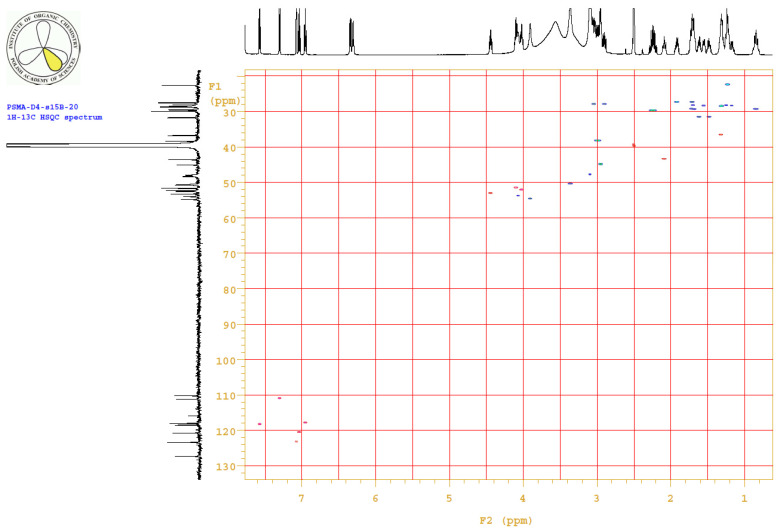
The ^1^H-^13^C HSQC spectrum of PSMA-D4 in DMSO at 298 K.

**Figure A10:**
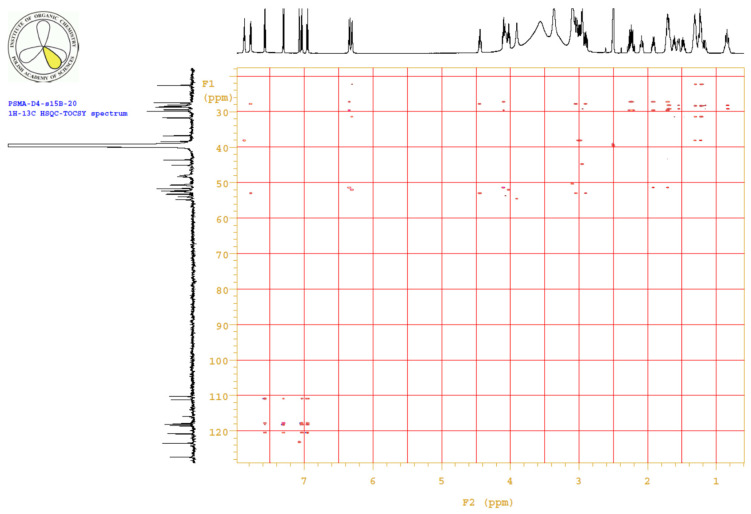
The ^1^H-^13^C HSQC-TOXY spectrum of PSMA-D4 in DMSO at 298 K.

**Figure A11:**
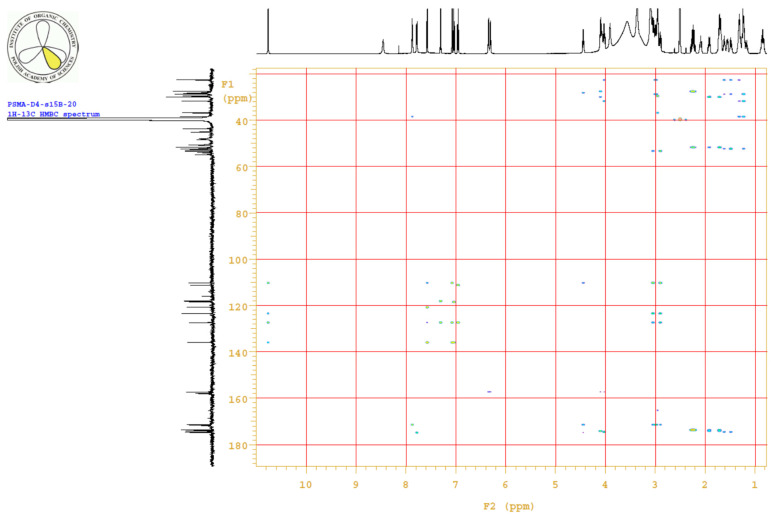
The ^1^H-^13^C HMBC spectrum of PSMA-D4 in DMSO at 298 K.

**Figure A12:**
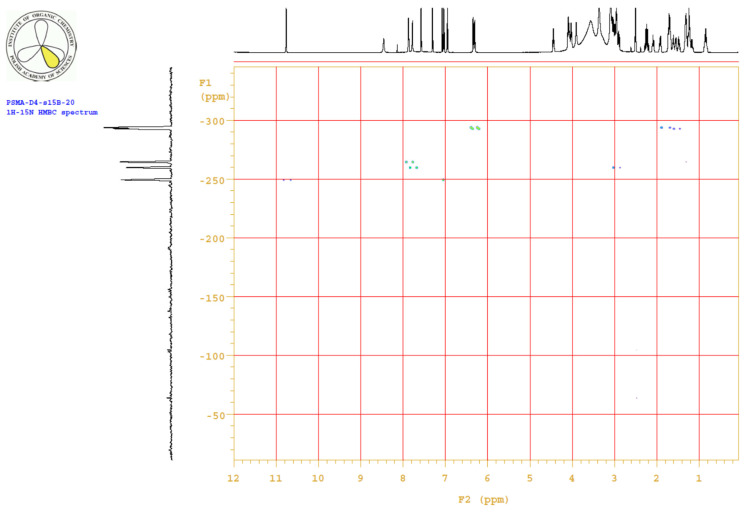
The ^1^H-^15^N HMBC spectrum of PSMA-D4 in DMSO at 298 K.

**Figure A13:**
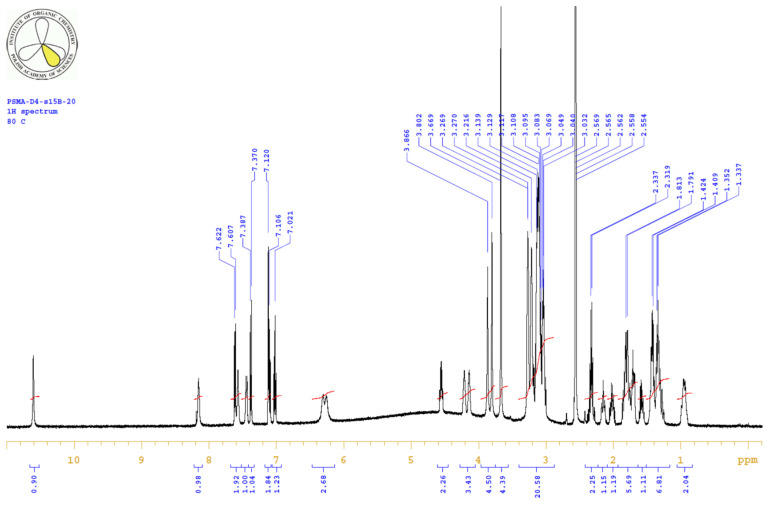
Full ^1^H NMR spectrum of PSMA-D4 in DMSO at 353 K.

**Figure A14:**
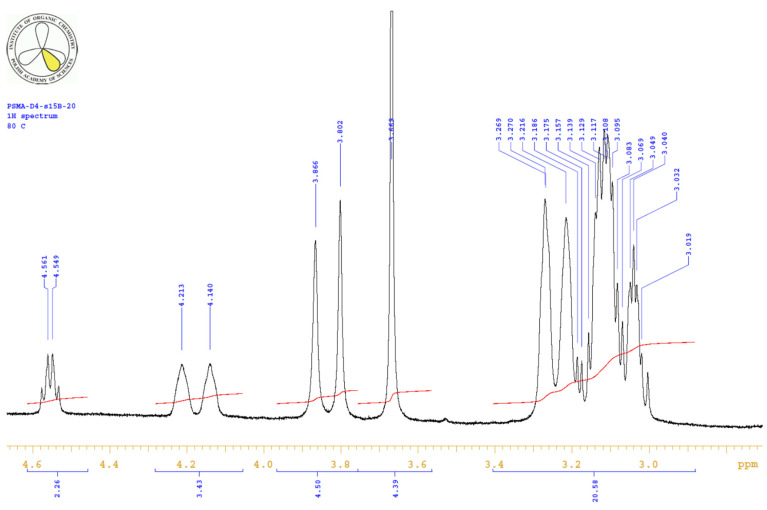
Up-field part of the ^1^H NMR spectrum of PSMA-D4 in DMSO at 353 K.

**Figure A15:**
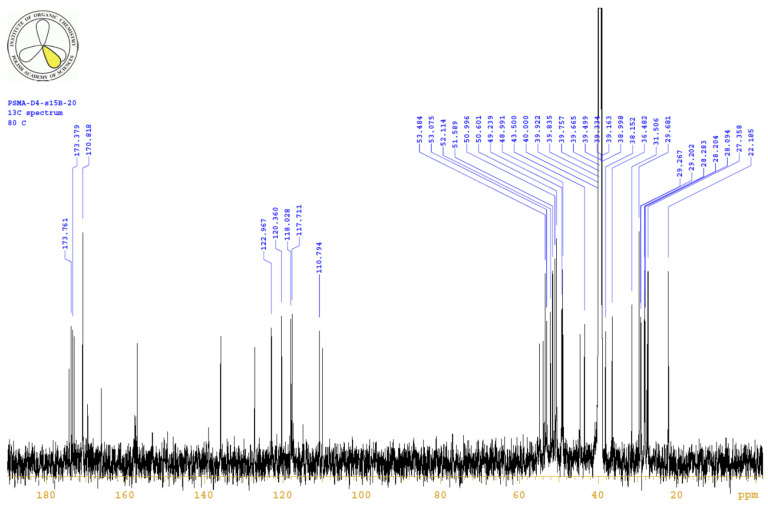
Full ^13^C NMR spectrum of PSMA-D4 in DMSO at 353 K.

**Figure A16:**
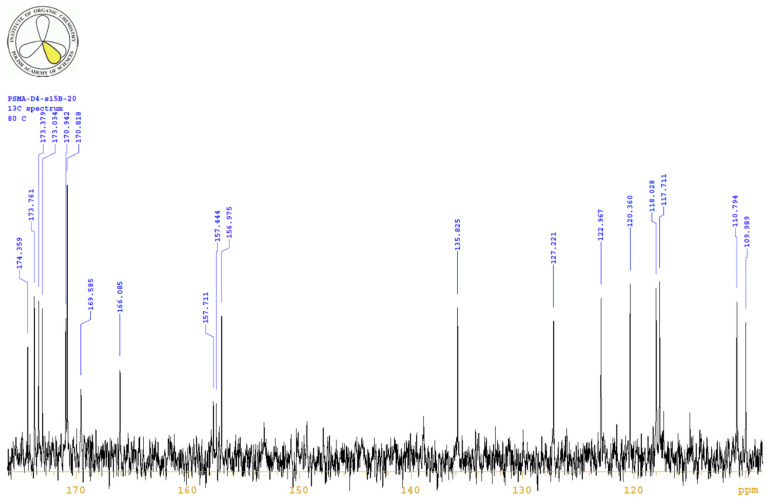
Up-field part of the ^13^C NMR spectrum of PSMA-D4 in DMSO at 353 K.

**Figure A17:**
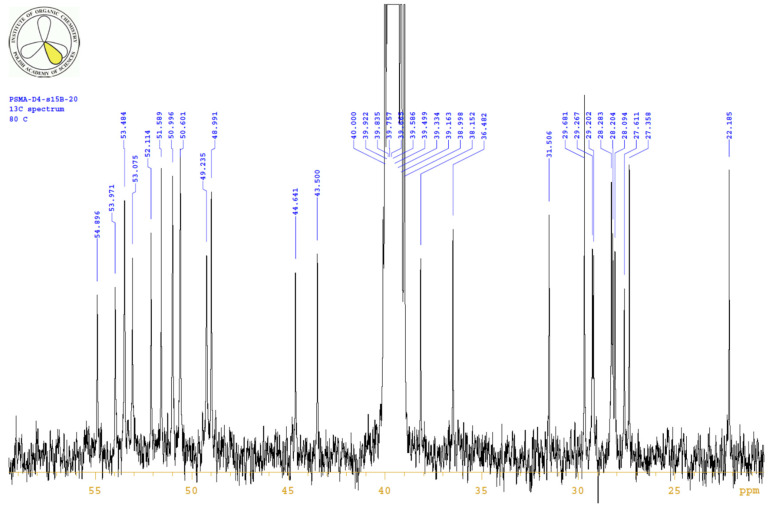
Down-field part of the ^13^C NMR spectrum of PSMA-D4 in DMSO at 353 K.

**Figure A18:**
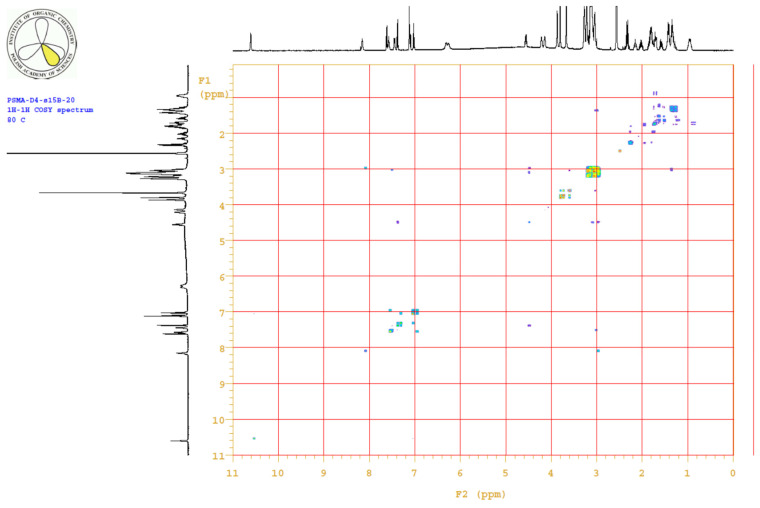
The ^1^H-^1^H COSY spectrum of PSMA-D4 in DMSO at 353 K.

**Figure A19:**
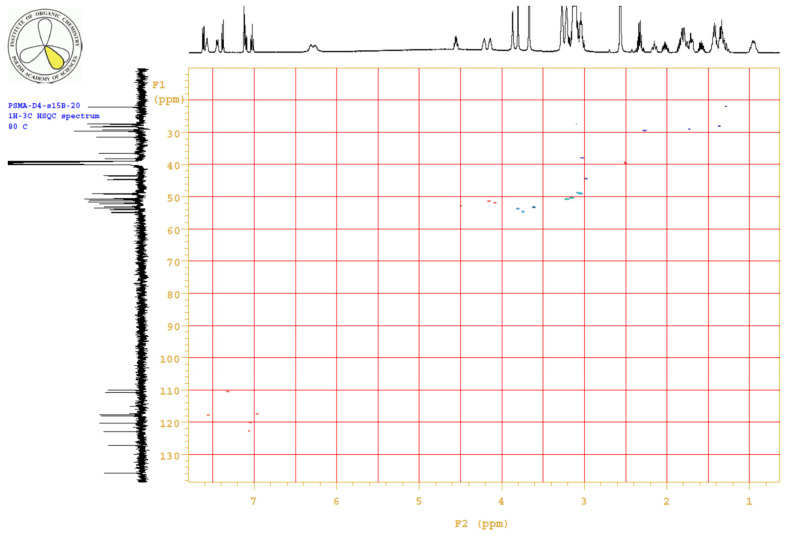
The ^1^H-^13^C HSQC spectrum of PSMA-D4 in DMSO at 353 K.

**Figure A20:**
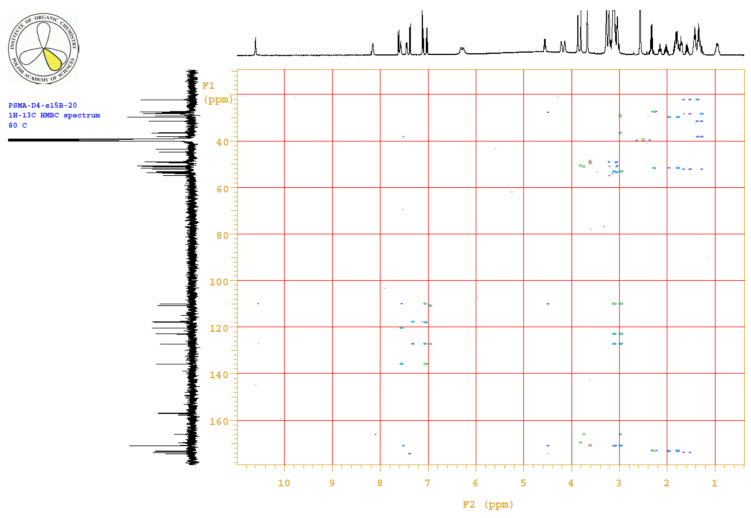
The ^1^H-^13^C HMBC spectrum of PSMA-D4 in DMSO at 353 K.

**Figure A24:**
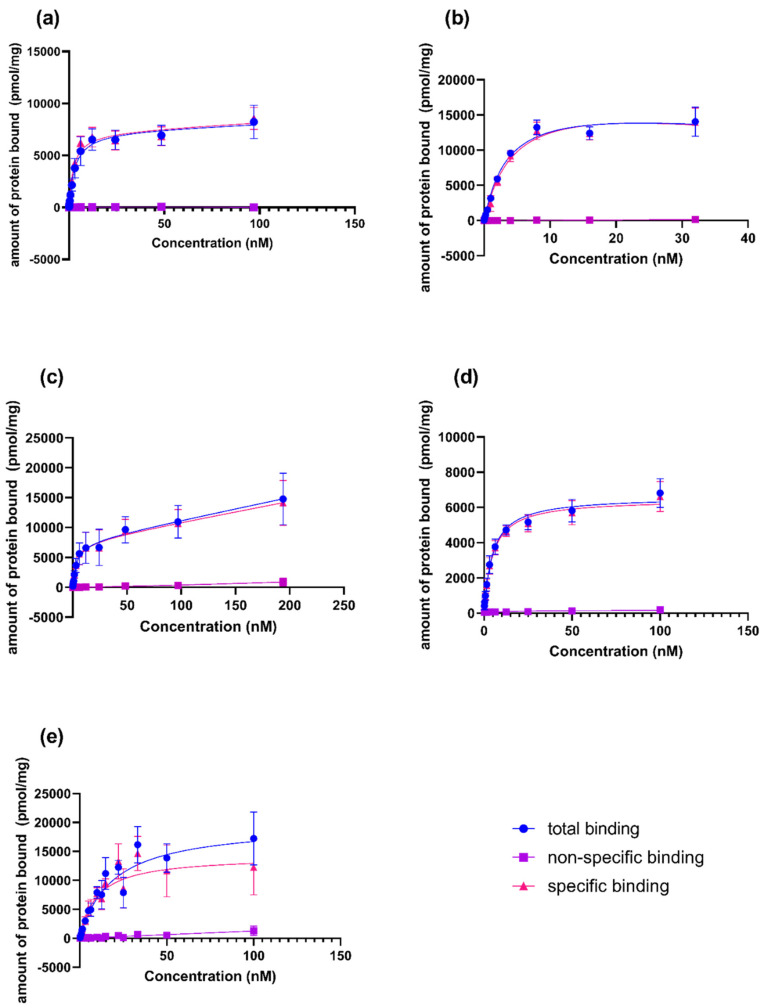
Saturation binding curve for PSMA inhibitors: (**a**)—[^177^Lu]Lu-PSMA-D4, (**b**)—[^90^Y]Y-PSMA-D4, (**c**)—[^47^Sc]Sc-PSMA-D4, (**d**)—[^177^Lu]Lu-PSMA I&T, and (**e**)—[^68^Ga]-PSMA 11. Total binding (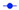) and non-specific binding (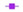) were determined by incubation PSMA ligands with LNCaP (PSMA positive) and PC3 (PSMA negative) membranes, respectively. The specific binding (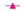) was evaluated as a difference between the total and non-specific binding.

## Data Availability

Not applicable.

## Abbreviations

PCa	Prostate Cancer
PSMA-D4	Prostate-Specific Membrane Antigen ligand conjugated with DOTA (Glu-CO-Lys-L-Trp-4-Amc-DOTA)
DOTA	1,4,7,10-Tetraazacyclododecane-1,4,7,10-tetraacetic acid, tetraxetan
mCRPC	metastatic castration-resistant prostate cancer
PSA	prostate-specific antigen
GCPII	glutamate carboxypeptidase II
BFC	bifunctional chelator
LNCaP	Lymph Node Carcinoma of the Prostate
COMU	1-[(1-(cyano-2-ethoxy-2-oxoethylideneaminooxy)dimethylaminemorpholino)]uronium hexafluorophosphate
K_D_	binging affinity
B_max_	maximum receptor density
CLI	Cerenkov luminescence imaging
ROI	region of interest
SA	specific activity
RCY	radiochemical yield
ATCC	American Type Culture Collection
FBS	fetal bovine serum
ID	injected dose
SD	standard deviation
SE	electrospray soure
IT	ion trap
TOF	time of flight analyzer
TCD	Thermal conductivity detector

## Appendix A

### Synthesis of PSMA-D4

The PSMA-D4 synthesis was started from the preparation of Glu(tBu)-urea-Lys-NH_2_ carried out on solid phase Wang polystyrene resin, crosslinked with divinyl-benzene, which is widely used in peptide synthesis. Fmoc-L-Lys(Alloc) was attached to hydroxyl groups in the support in the manner described previously [52].

After the reaction had been completed, the support was washed with N,N-dimethylformamide, 50% N,N-dimethylformamide solution in dichloromethane, and dichloromethane and dried in vacuum.

The amino acid loading was measured using a method described by Eissler et al., [53].

The Fmoc protection group was removed in the manner usually used in peptide synthesis [52].

A double molar excess of triphosgene in dry dichloromethane was cooled in a dry-ice bath to ≤−50 °C. The solution of L-Glu(tBu)OtBu*HCl 0.75% N,N-diisopropylethylamine in dry dichloromethane was prepared separately using sixfold molar excess. The solution was slowly added dropwise into triphosgene solution with stirring not to exceed −50 °C. After the solution was added, the bath was removed, and the solution was stirred until it reached room temperature. To the isocyanate solution prepared, dried resin with L-Lys(Alloc) was added, and the mixture was stirred overnight. The resin was filtered, washed with dichloromethane, and dried in vacuum.

The reaction of Alloc group detachment was performed in darkness. In the dark, glass vessel the following solution was prepared: 0.1 × molar excess of (catalyst) Pd[P(Ph)3]4 (tetrakis(triphenylphosphine)palladium(0)) in 10% solution of morpholine in dry dichloromethane. The resin was swollen in dichloromethane and stirred 3 × 1 h each time with the new solution described above. The resin was filtered off and washed with N,N-dimethylformamide, 2% N,N-diisopropylethylamine in N,N-dimethylformamide, the solution of 20 mg mL^−1^ sodium diethylotiokarbamate in N,N-dimethylformamide, N,N-dimethylformamide, and dichloromethane. It was dried under vacuum.

Further synthesis steps on solid support were performed in an automatic peptide synthesizer LibertyBlue equipped with Discover microwave oven (CEM). The microwave radiation speeds up the reaction, especially when coupling bulky chelators like DOTA [42,43].

1-[(1-(cyano-2-ethoxy-2-oxoethylideneaminooxy)dimethylaminemorpholino)]uronium hexafluorophosphate (COMU) was used as a coupling reagent for automated synthesis on the resin, with N,N-diisopropylethylamine as a base [44]. Amino acids Fmoc-l-Trp(Boc) and Fmoc-4-Amc were coupled to Glu(tBu)-urea-Lys-NH_2_ moiety prepared on the resin using standard coupling program of the synthesizer.

The chelator DOTA(tBu)_3_ required an extended reaction time in milder conditions; it was coupled at room temperature for 1 h following microwave heating to 50 °C for 30 min. The microwave-assisted synthesis is, in this case, fast. It needs to be performed immediately after preparing the reagents solutions due to the instability of COMU and Fmoc-4-Amc over a long time [54].

The product was detached from the resin and deprotected by stirring with the solution of 2% of triisopropylsilane, 2% of phenol, 2% of water, 2% of thioanisole, and 2% of 1,2-ethanedithiol in trifluoroacetic acid for 6 h, based on the publication of Wängler et al. [45]. The resulting product was precipitated and washed with diethyl ether. The precipitate was washed with diethyl ether through centrifugation. The precipitate was dissolved in 0.1% solution of trifluoroacetic acid in water and heated up on a rotary evaporator to 60 °C at 800 mbar for 1 h to detach any remaining Boc groups from tryptophane. The solution was frozen and lyophilized.

## Appendix B

### Appendix B.1. Physico-Chemical Characteristics of PSMA-D4

The NCNR and multinuclear MR spectra were performed in DMSO-D6 solution at two different temperatures (298 and 353 K) to confirm the structure of the compound supplied, labeled as PSMA-D4-s15-20, Scheme A3.

### Appendix B.2. Equipment and Reagents

#### Appendix B.2.1. Instruments

The NMR spectra were recorded at Varian VNMRS-600 and VNMRS-500 spectrometers equipped with a 5 mm PFG AutoXID (^1^H/X ^15^N-^31^P) and 5 mm PFG AutoXDB (^1^H-^19^F/X ^15^N-^31^P) probes, respectively.

#### Appendix B.2.2. Reagents and Sample Preparation

The sample of ca. 20 mg of the compound under investigation was dissolved in 0.6 ml of DMSO-D6—99.80 %D, Euriso-top lot: T1121, batch: 0320C.

### Appendix B.3. Methodology

At first 1D/2D NMR spectra were measured at 298 K in deuterated dimethyl sulfoxide (DMSO). They were calibrated using appropriate signals of DMSO (δ = 2.50 ppm (^1^H NMR), and δ = 39.5 ppm (^13^C NMR)) as standard for ^1^H/^13^C NMR spectra, respectively.

Due to a partial broadening of ^1^H NMR signals at 298 K in range δ ca. 4–2.7 ppm and water presence, sequence to remove broad signals was used for simplifying ^1^H NMR spectra and better presentation of them. Relatively broadened ^1^H signals at T = 298K related to 1,4,7,10-tetrakis(carboxymethyl)-1,4,7,10-tetraazacyclododecane (ring A) hindered the assignments of the ^1^H and ^13^C signals of the ring A, and that is why higher temperature measurements (T = 353 K) were also performed.

The structure of the compound (Scheme 1) was determined by interpretation of the one-dimensional ^1^H and ^13^C, two-dimensional homonuclear COSY and TOCSY, and heteronuclear ^1^H-^13^C HSQC, HSQC-TOCSY, and HMBC NMR spectra. Proton connectivities were derived from COSY and TOCSY spectra. The ^13^C resonances corresponding to carbons with directly attached protons were assigned using HSQC and HSQC-TOCSY spectra. HMBC correlations were used to assign resonances of the quaternary carbons and to validate the connectivities established by the other spectra. Results of ^1^H-^15^N correlations HMBC were used for confirmation of character of nitrogen atoms in the compound studied.

The assignment of the ^1^H and ^13^C signals of part of the molecule (chain C1–C36) was made twice, whereas assignment of the ^1^H and ^13^C signals of the ring A was made only at 353 K. The ^15^N spectrum (performed at 298 K only) was calibrated using nitromethane as external standard for which δ = 0.0 ppm, and the ^15^N chemical shifts were determined on the basis of 2D ^1^H-^15^N HMBC spectrum. The NMR data for both temperatures were presented in Table A1.

**Table A1:** NMR signals assignment.

PositionNumber	δ (15N) * [ppm]	δ (13C) ** [ppm]		δ (1H) ** [ppm]		Multiplicity (a)	(H,H) Couplings [Hz]
	298 K	298 K	353 K	298 K	353 K	298 K	298 K
1	-----	173.7	173.0	-----	-----	-----	
OH (C1)	-----	-----	-----	~12.0 b	c	s (br) d	
2	-----	29.9	29.7	2.23	2.26	m	5.7, 9.0, 16.3
3	-----	27.5	27.4	1.70/1.91	1.76/1.95	m(ov)/m	
4	-----	51.6	51.6	4.08	4.14	m	
5	-----	174.2	173.4	-----	-----	-----	
OH (C5)	-----	-----	-----	~12.0 b	c	s (br) d	
6	−294.0	-----	-----	6.34	6.23 e	d	8.4
7	-----	157.3	157.0	-----	-----	-----	
8	−292.9	-----	-----	6.30	6.20 e	d	8.2
9	-----	52.4	52.1	4.01	4.07	m	
10	-----	174.6	173.8	-----	-----	-----	
OH (C10)	-----	-----	-----	~12.0 b	c	s (br) d	
11	-----	31.7	31.5	1.48/1.61	1.51/1.64	m/m	
12	-----	22.6	22.2	1.22	1.27	m(ov)	
13	-----	28.7	28.3	1.30	1.35	m(ov)	
14	-----	38.4	38.2	2.98	3.00	m(ov)	
15	−264.6	-----	-----	7.86	7.50	t	5.9
16	-----	171.4	170.9	-----	-----	-----	
17	-----	53.3	53.1	4.44	4.48	m	
18	-----	28.1	27.6	2.89/3.04	2.95/3.09	dd/m(ov)	8.7, 14.5/---
19	-----	110.3	110.0	-----	-----	-----	
20	----	123.5	123.0	7.07	7.05	d	2.2
21	−249.5	-----	-----	10.75	10.54	s	
22	-----	136.0	135.8	-----	-----	-----	
23	-----	111.2	110.8	7.29	7.30	ddd	0.9, 1.2, 8.2
24	-----	120.8	120.4	7.03	7.03	ddd	1.1, 6.9, 8.2
25	-----	118.1	117.7	6.94	6.95	ddd	1.0, 7.0, 7.8
26	-----	118.5	118.0	7.56	7.54	d	7.8
27	-----	127.4	127.2	-----	-----	-----	
28	−269.9	-----	-----	7.77	7.37	d	8.3
29	-----	174.9	174.4	-----	-----	-----	
30	-----	43.6	43.5	2.08	2.08	m	
31	-----	28.6	28.2	1.16/1.54	1.22/1.62	m(ov)/m(ov)	
32	-----	29.6	29.2	0.82/1.67	0.90/1.73	m(ov)/m(ov)	
33	-----	37.8	36.5	1.32	1.35	m(ov)	
34	-----	29.5	29.3	0.84/1.71	0.88/1.71	m(ov)/m(ov)	
35	-----	28.4	28.1	1.24/1.69	1.27/1.73	m(ov)/m(ov)	
36	-----	45.1	44.6	2.94	2.96	m(ov)	
37	f	-----	-----	8.45	8.08	s(br)	
38	-----	165.3	166.1	-----	-----	-----	
39	-----		54.9		3.72	-----	
40	f	-----	-----	-----	-----	-----	
41/57	-----		51.0		3.19	-----	
42/56	-----		49.0		3.05	-----	
43/53	f	-----	-----	-----	-----	-----	
44/54	-----		53.5		3.59	-----	
45/55	-----	-----	170.8	-----	----	-----	
46/52	f	-----	49.2		3.03	-----	
47/51	-----		50.6		3.14	-----	
48	-----		-----	-----	-----	-----	
49	f	-----	54.0		3.79	-----	
50	-----		169.6		-----	-----	
OH g	-----		-----	~12.0 b	c	-----	

*—^15^N measurements were performed at 298 K only; **—assignment of the ^1^H/^13^C signals of ring A at room temperature (298 K) is uncertain, ^1^H NMR broad signals are at δ ca. 3.88, 3.53, 3.34, and 3.07 ppm, whereas ^13^C NMR broad signals are at δ ca. 54.8, 54.0, 52.5, 50.7, 50.6, 48.3, and 48.0 ppm; a—multiplicity: [s—singlet, d—doublet, dd—doublet of doublets, ddd- doublet od doublets of doublets, t—triplet, m—multiplet, m(ov)—overlapping multiplets s(br)—broad singlet]; b—broad averaged signal for all protons of COOH groups; c—signals not observed due to fast exchange of all COOH protons at higher temperature; d—very broad averaged singlet; e—low intensity of ^1^H signals due to exchange and broadening at higher temperature, f—not recorded in the ^1^H-^15^N HMBC experiments, most probably due a signal width related to exchange process; g—^1^H NMR signals of other COOH groups at C44, C49, and C54 are averaged at 298 K and very broad (not observed) at 353 K.

The experiments were performed in the following conditions:

^1^H spectra—64 transients, relaxation delay: 1.0 s, pulse width: 2.3 µs (30⁰), 64 K data points zero-filled to 128 K, and spectral width ca. 10,000 Hz.

{^1^H}^13^C NMR—30,000 transients, relaxation delay: 0.5 s, pulse width:4.2 µs (30⁰), 90 K data points zero-filled to 128 K, and spectra width ca. 38,000 Hz.

COSY—spectral widths 7200 Hz in both dimensions, 512 complex point in *t_2_* and *t_1_*, two scans per increment, and relaxation delay 1s.

TOCSY—spectral widths 7200 Hz in both dimensions, 512 complex points in *t_2_* and *t_1_*, two scans per increment, relaxation delay 1s, and spin-lock time 80 ms, respectively.

^1^H-^13^C HSQC—spectral widths 7200 Hz in F2 and 27,000 Hz in F1 (^13^C), 1024 complex points in *t_2_*, 2048 complex points in *t_1_*, 2 scans per increment, and relaxation delay 1s.

^1^H-^13^C HSQC-TOCSY—spectral widths 7200 Hz in F2 and 27,000 Hz in F1, 1024 complex points in *t_2_*, 2048 complex points in *t_1_*, two scans per increment, relaxation delay 1 s, and spin-lock time 80 ms.

^1^H-^13^C/(^1^H-^15^N) HMBC—spectral widths 7200 Hz in F2 and 27,000 Hz in F1 (^13^C) or 22,000 Hz (^15^N), 1024 complex points in *t_2_*, 2048 complex points in *t_1_*, 16 or 32 (^15^N) scans per increment.

The data were processed with linear prediction in t1 followed by zero-filling in both dimensions. Gaussian weighting functions were applied in both domains prior to Fourier transformation. In the cases where the signal to noise was sufficient, the use of sine weighting functions facilitated a better resolution of the spectra.

In Figure A1, Figure A2, Figure A3, Figure A4, Figure A5, Figure A6, Figure A7, Figure A8, Figure A9, Figure A10, Figure A11, Figure A12, Figure A13, Figure A14, Figure A15, Figure A16, Figure A17, Figure A18, Figure A19 and Figure A20, all basic spectra and some important expansions for recoded experiments are presented.

### Appendix B.4. Results

A careful analysis of the results from various NMR spectra (298 and 353 K) confirms the compound studied structure shown in Scheme 1. However, ^1^H and ^13^C signals coming from ring A at room temperature (298 K) are significantly broadened due to the exchange process. Application of higher temperature (353 K) made this process faster, and NMR signals are thinner/narrower, allowing their assignment to proper atoms/nuclei. Half-height widths in the case of proton signals of ring A (at room temperature and 353 K) were still too big to make ^1^H–^15^N transfer possible, and that is why no ^15^N signals for ring A were observed in ^1^H-^15^N HMBC experiment. Additionally, in the long-accumulated ^13^C NMR spectrum of compound supplied, a quartet at 158.5 ppm (*^2^J_C-F_* = 32 Hz) was observed, suggesting the presence of trifluoroacetic acid in the sample.

## Appendix D

In vitro results
